# Core outcome set for early intervention trials to prevent obesity in childhood (COS-EPOCH): Agreement on “what” to measure

**DOI:** 10.1038/s41366-022-01198-w

**Published:** 2022-08-04

**Authors:** Vicki Brown, Marj Moodie, Marufa Sultana, Kylie E. Hunter, Rebecca Byrne, Anna Lene Seidler, Rebecca Golley, Rachael W. Taylor, Kylie D. Hesketh, Karen Matvienko-Sikar

**Affiliations:** 1 Deakin University, Deakin Health Economics, Institute for Health Transformation, Global Obesity Centre (GLOBE), School of Health and Social Development, Geelong, VIC 3220 Australia; 2 Centre for Research Excellence in the Early Prevention of Obesity in Childhood, University of Sydney, Sydney, NSW Australia; 3 NHMRC Clinical Trials Centre, University of Sydney, Sydney, NSW Australia; 4 Queensland University of Technology, School of Exercise and Nutrition Sciences, Brisbane, QLD Australia; 5 Caring Futures Institute, College of Nursing and Health Sciences, Flinders University, Adelaide, SA 5042 Australia; 6 Department of Medicine, University of Otago, Dunedin, New Zealand; 7 Deakin University, Institute for Physical Activity and Nutrition, Geelong, VIC 3220 Australia; 8 School of Public Health, University College Cork, Cork, Ireland

**Keywords:** Lifestyle modification, Risk factors, Paediatrics

## Abstract

**Background:**

Heterogeneity in the outcomes collected and reported in trials of interventions to prevent obesity in the first five years of life highlights the need for a core outcome set to streamline intervention evaluation and synthesis of effects. This study aimed to develop a core outcome set for use in early childhood obesity prevention intervention studies in children from birth to five years of age (COS-EPOCH).

**Methods:**

The development of the core outcome set followed published guidelines and consisted of three stages: (1) systematic scoping review of outcomes collected and reported in early childhood obesity prevention trials; (2) e-Delphi study with stakeholders to prioritise outcomes; (3) meeting with stakeholders to reach consensus on outcomes. Stakeholders included parents/caregivers of children aged ≤ five years, policy-makers/funders, researchers, health professionals, and community and organisational stakeholders interested in obesity prevention interventions.

**Results:**

Twenty-two outcomes from nine outcome domains (anthropometry, dietary intake, sedentary behaviour, physical activity, sleep, outcomes in parents/caregivers, environmental, emotional/cognitive functioning, economics) were included in the core outcome set: infant tummy time; child diet quality, dietary intake, fruit and vegetable intake, non-core food intake, non-core beverage intake, meal patterns, weight-based anthropometry, screentime, time spent sedentary, physical activity, sleep duration, wellbeing; parent/caregiver physical activity, sleep and nutrition parenting practices; food environment, sedentary behaviour or physical activity home environment, family meal environment, early childhood education and care environment, household food security; economic evaluation.

**Conclusions:**

The systematic stakeholder-informed study identified the minimum outcomes recommended for collection and reporting in early childhood obesity prevention trials. Future work will investigate the recommended instruments to measure each of these outcomes. The core outcome set will standardise guidance on the measurement and reporting of outcomes from early childhood obesity prevention interventions, to better facilitate evidence comparison and synthesis, and maximise the value of data collected across studies.

## Introduction

Childhood obesity is a global concern, with an estimated 39 million children under the age of five living with overweight or obesity in 2020 [1]. Short-term physical and psychological health problems associated with obesity in childhood include asthma, sleep apnoea, high blood pressure and lower health-related quality of life [2, 3]. Obesity in childhood is a strong predictor of obesity in adulthood, with the associated risk for a number of conditions, including type 2 diabetes, some cancers and cardiovascular disease [1]. Given the high prevalence of overweight or obesity in young children, there is a clear need for early intervention, to ensure children attain and maintain a healthy growth trajectory during the early years and to reduce the huge social, health and economic burden [4].

A growing number of early childhood obesity prevention trials are being conducted worldwide [5]. However heterogeneity in the outcomes collected and reported in efforts to synthesise the study findings of these interventions is hindering the understanding of what interventions work best, for whom and through which mechanisms [6]. Core outcome sets (COS) are agreed minimum sets of outcomes recommended for measurement and reporting in studies in specific health areas to allow cross study comparison of findings [7]. The development and use of COS can reduce outcome heterogeneity and research waste, and improve evaluation and synthesis of intervention effects. Further, the process of COS development involves key stakeholders at all stages, thus ensuring that COS are relevant and appropriate for specific health areas in which they will be used [7].

A COS has been developed to identify a minimum outcome set for infant feeding interventions; [8, 9] it identified twenty-six outcomes, across nine outcome domains that should be reported in trials of interventions targeting infant feeding as a strategy to prevent obesity in children ≤ 1 year old [8]. Given the complex and multi-faceted aetiology of childhood obesity [10], the development of a COS that can be more broadly applied to prevention interventions for children aged from birth to five years and that span a wider range of risk factors is warranted [11]. Considering a broader range of outcomes is important, given that multiple long-term behaviours are established in the infancy and preschool period of a child’s life, and interventions often incorporate multiple behaviour targets. This study therefore aimed to develop the Core Outcome Set for Early intervention trials to Prevent Obesity in CHildhood (COS-EPOCH); for trials of early childhood obesity prevention interventions in children aged from birth to five years, with lifestyle-related components targeting a broad range of relevant risk factors (e.g. diet, physical activity, sedentary behaviour, sleep, parent/caregiver practices) and conducted across a range of relevant settings (e.g. community, home, early childhood education and care [ECEC]).

## Methods

The study is registered on the Core Outcome Measures in Effectiveness Trials (COMET) Initiative registry of COS (registration number 1679, http://www.comet-initiative.org/Studies/Details/1679). Development of the COS followed the Core Outcome Set-STAndards for Development (COS-STAD) recommendations [7, 12]. Reporting of the COS follows the Core Outcome Set-STAndards for Reporting (COS-STAR Statement; Supplementary Table S1) [13].

The study was undertaken between June 2020 and March 2022 in three stages:

Stage 1 - A systematic scoping review of randomised controlled trials (RCTs) of early childhood obesity prevention interventions to identify outcomes reported in existing trials; [14]

Stage 2- An e-Delphi study to elicit views on the importance of potential COS outcomes from key stakeholders;

Stage 3- An online consensus meeting with key stakeholders to finalise the core outcome set.

An international Steering Group, chaired by the lead author (VB) and comprised of eight experts in the field of early childhood obesity prevention interventions, provided expert oversight and guided the development of the COS in all stages. Steering Group members were invited via the lead author’s professional networks, and membership to the Steering Group was designed to incorporate expertise across the range of risk factors and settings pertinent to early childhood obesity prevention intervention. Steering Group members had expertise in health economics, psychology, pediatric public health, behavioural epidemiology, public health nutrition, physical activity and sedentary behaviour, sleep, biostatistics and evidence synthesis. Ethical consent was obtained from the Deakin University Human Research Ethics Committee (HEAG-H 231_2020).

### Systematic scoping review

In order to develop the COS-EPOCH, a systematic scoping review was first undertaken to identify commonly collected and reported outcomes from early childhood obesity prevention interventions [11, 14]. A brief overview of the scoping review methods is provided here, with further details available in the full publication [14]. Publicly available clinical trial registries (clinicaltrials.gov and via the World Health Organisation International Clinical Trials Registry Platform (WHO ICTRP)) and Ovid Medline were systematically searched, using pre-defined search strategies [14]. The clinical trial registry search was supplemented by a review and update of a recently published Cochrane review including obesity prevention intervention RCTs in children aged under five years [5].

Studies were included if they were RCTs of interventions starting antenatally to child age five years and aimed to prevent childhood overweight or obesity, with a component related to lifestyle (e.g., diet, physical activity, sedentary behaviour, sleep, parent/caregiver practices). Potential studies were screened by two independent reviewers (VB, MS), and a data extraction tool was developed in Microsoft Excel following COMET recommendations [7]. A deductive iterative approach was followed to categorise the conceptualisation of outcome domains and outcomes and reach agreement on final outcomes and definitions, with the participation and agreement of all Steering Group members. Findings of the review are available elsewhere [14].

### e-Delphi study

A three-round e-Delphi survey was conducted to achieve convergence of opinion from key stakeholders on the importance of outcomes identified in the scoping review, following published guidelines (Supplementary Table S2) [7, 15]. Given the potential for a large number of outcomes to be identified in the scoping review, and recent evidence suggesting that Delphi studies that included a higher number of items had significantly lower response rates [16], decision rules for inclusion of outcomes into the e-Delphi survey were developed a priori in agreement with the Steering Group. Outcomes were omitted if they were dietary intake or feeding outcomes from interventions in infants aged ≤ 1 year, as the COS-EPOCH is designed to be complementary to the published COS for early infant feeding interventions [8]. Outcomes were also omitted for inclusion into the e-Delphi survey if they were not clearly specified (for example, reported as ‘changes in health behaviours’), study-related (for example, feasibility or fidelity outcomes) or if the scoping review results suggested they were included in only one of the 161 included studies [14]. To further reduce the burden on e-Delphi study participants, several outcomes were merged (for example, ‘height’ and ‘weight’ were merged with ‘body mass index’ into ‘weight-based anthropometry’), following consultation and full agreement with the Steering Group.

Early childhood obesity prevention intervention stakeholder groups were identified in conjunction with the Steering Group as: (i) policy-makers/funders, (ii) parents/caregivers, (iii) researchers, (iv) health professionals, and (v) community and organisational stakeholders interested in obesity prevention interventions (for instance, representatives from settings where interventions are undertaken such as Maternal Child Health centres, ECEC, health promotion organisations). Although there is no consensus on the number of participants or rounds required for a Delphi study [17, 18], the study aimed to recruit a sample size of 30 participants per stakeholder group (*n* = 150). This sample size was based on a recent study investigating attrition between two rounds of Delphi surveys that estimated that the overall response rate ranges from 45% to 100%, but is typically 80% or higher [16]. Assuming attrition of between 20% and 55%, this would result in between 9 and 19 participants per stakeholder group at the end of three rounds (approximately 45–96 full completions by the end of round three).

Potential study participants were recruited over a four-week period, from 27 August 2021. A four-week sponsored social media campaign using Facebook and Instagram invited parents/caregivers to click on a link to learn more about the study and participate after giving consent. Inclusion criteria for parents/caregivers included having at least one child aged from birth to five years; being fluent in English; and being able to freely give informed consent. A list of potential participants from all other stakeholder groups (policy-makers/funders, researchers, health professionals, and community and organisational stakeholders) was compiled using internet sources, the published literature and publicly available information. Potential researcher participants were identified by searching Scopus and Google Scholar databases for publications using relevant keywords related to early childhood obesity prevention, and sourcing author contact details either from academic papers or institutional websites. Health professional contacts were also sourced using academic literature, the websites of relevant conferences and professional organisations. Publicly available websites for ECEC settings, health promotion and advocacy organisations were used to identify potential community and organisational stakeholders for obesity prevention interventions. Potential policy-maker and funder participants were identified through searches of relevant government websites (e.g., Departments of Health), philanthropic organisations and the World Health Organization European Childhood Obesity Surveillance Initiative (WHO COSI) website. Personalised emails with study information were sent to all potential participants, with a follow-up email sent after seven days if no response was received. Participants were invited to forward study details on to any colleagues they thought may be interested to contribute to the study.

Outcomes were presented in the e-Delphi questionnaire by outcome domain, with the ordering of domains (and outcomes per domain) randomised to minimise response bias; a plain language definition of each outcome was provided. The questionnaire was pilot tested with the Steering Group and a convenience sample of non-academic parents/caregivers (*n* = 7) prior to study launch, and feedback to improve the comprehension of the study aims and definitions of outcomes was incorporated. Participants in the e-Delphi were asked to rate the importance of each outcome based on a Likert scale anchored between 1 and 9 based on GRADE, where 1–3 signifies an outcome that is ‘not that important’, 4–6 ‘important but not critical’ and 7–9 ‘critically important’ [19]. At the end of round 1, participants were also asked to list any additional outcomes they felt should be included in the survey and these were considered for inclusion in round 2 by the Steering Group.

Responses were analysed both within and between groups of stakeholders (group mean, median, strength of agreement using mean absolute deviation from the median (MADM) [20]). Levels of agreement using the MADM were defined using values from the literature (low > 1.41; moderate 1.08–1.41; high < 1.08) [20] and criteria for inclusion and exclusion of outcomes was pre-defined [21, 22] (Table 1). During subsequent rounds of the survey, participants received a summary of their individual responses for the previous round and a graphical depiction of the distribution of scores by stakeholder group. An example of this graphical feedback from round 1 of the study is given in Supplementary Fig. S1. Participants were then invited to review and re-rate outcomes. Outcomes that reached consensus to not be included as a core outcome by the end of round 2 were not brought forward to round 3. Attrition bias was examined to explore whether participant withdrawal from later rounds could be attributed to holding views not shared by the majority of stakeholder group peers [23]. This was done by analysing the mean response and response distributions across all outcome scores between participants completing only round 1 of the survey with participants who completed all rounds [7, 23]. The e-Delphi was conducted in DelphiManager Software and all participants provided consent to participate before being directed to the round 1 survey [24].

**Table 1:** Outcome inclusion and exclusion criteria.

Consensus to include as a core outcome	≥ 75% of participants in each stakeholder group score the outcome as ‘critically important’ AND < 15% of participants in each stakeholder group score the outcome as ‘not that important’.
Consensus to not include as a core outcome	≥ 75% of participants in each stakeholder group score the outcome as ‘not that important’ AND < 15% of participants in each stakeholder group score the outcome as ‘critically important’.
No consensus	All other combinations

### Consensus meeting

The results of the e-Delphi were presented to the Steering Group in February 2022, and feedback was provided on the recruitment and preparation for the consensus meeting to finalise the COS. Participants who completed round 3 of the e-Delphi survey were invited by email to participate in a half-day virtual consensus meeting held in March 2022. The consensus meeting utilised a nominal group technique (NGT) to discuss the results of the e-Delphi survey and to agree on a final COS [7]. NGT was selected as it enables consensus in a way that emphasizes the consideration of all participants’ views equally [25]. While a NGT does not depend on statistical power [26], we aimed to recruit a minimum of two and a maximum of four representatives from each stakeholder group to participate in the meeting (i.e., between ten and twenty participants total).

The consensus meeting was chaired by an experienced, external facilitator who was not known to any of the participants. Prior to the meeting, participants were emailed a guide to the consensus meeting, which outlined meeting access requirements, a point of contact for trouble-shooting, and a summary of the COS process and the NGT technique, based on the COMET Core Outcome Set and NGT plain language summaries [27].

At the start of the meeting the background and aims of the study and a lay definition of a COS were presented. Next, participants were presented with, and then discussed, the outcomes for which consensus for inclusion and exclusion had been reached in the e-Delphi process. Outcomes which had not reached consensus in the e-Delphi were then presented, and participants voted each outcome as ‘yes’ or ‘no’ for inclusion in the COS. Voting was undertaken electronically and anonymously, using Mentimeter [28]. The results of the first round of voting were briefly discussed before participants voted a second and final time. Outcomes which reached consensus to include (≥ 75% or more of participants rated as ‘yes’ for inclusion) were presented and discussed a final time, to finalise the COS. A final vote on whether the COS was agreed to and supported by all participants was then undertaken.

## Results

### Systematic scoping review

**Fig. 1:**
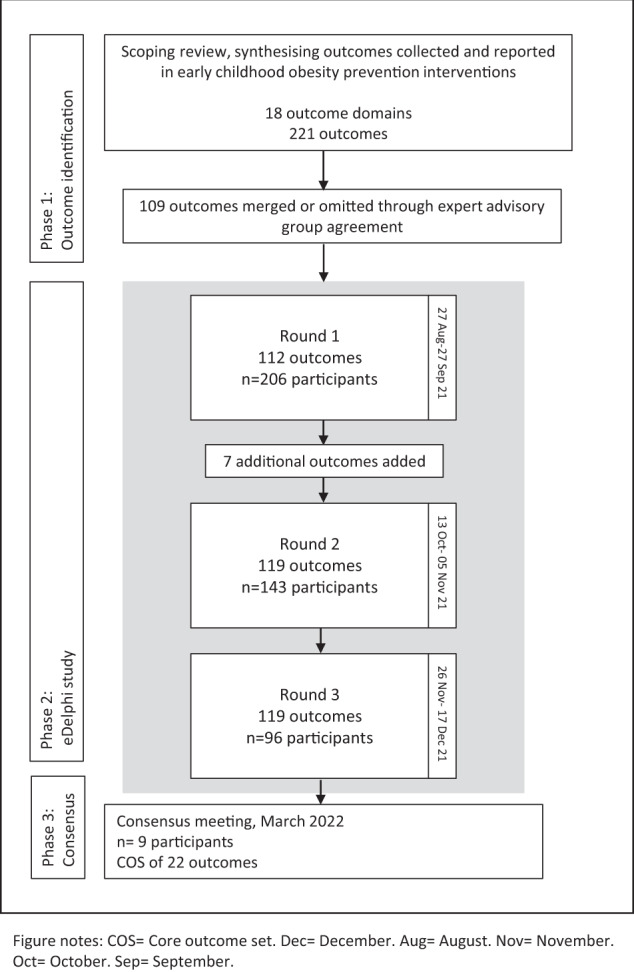
COS EPOCH development process.

The scoping review identified 18 outcome domains, from a total of 161 included studies: ‘anthropometry’, ‘dietary intake’, ‘feeding’, ‘physical activity’, ‘sedentary behaviour’, ‘sleep’, ‘perceptions and preferences’, ‘motor skill development’, ‘emotional functioning/wellbeing’, ‘cognitive/executive functioning’, ‘parenting practices’, ‘environmental’, ‘blood and lymphatic system’, ‘oral health’, ‘quality of life’, ‘economic’, ‘study-related’, ‘other’ [14]. In these domains, 221 unique outcomes were identified [14], representing an unrealistic number of outcomes for inclusion in the e-Delphi process given the potential for high participant burden. Following consultation and agreement with the Steering Group based on the a-priori decision rules, a total of 112 outcomes were included in the first round of the e-Delphi survey (Fig. 1; Supplementary Table S3).

### e-Delphi study

Round 1 of the e-Delphi study opened in August 2021, with 206 participants from 25 different countries (Table 2). A summary of e-Delphi results by stakeholder group and round are presented in Supplementary Table S4. In round 1, five outcomes reached consensus for inclusion in the COS: ‘child time spent sedentary’, ‘child physical activity’, ‘child diet quality’, ‘child dietary intake’ and ‘household food security’.

**Table 2:** Participant characteristics per Delphi round.

	Round 1	Round 2	Round 3
	*n*	*n* (response rate R1)	*n* (response rate R2)
**Stakeholder group**			
Researchers	85	69 (81%)	52 (75%)
Parents or caregivers	32	14 (44%)	8 (57%)
Health professionals	47	33 (70%)	18 (55%)
Policy-makers or funders	20	16 (80%)	11 (69%)
Community or organisational stakeholders	22	11 (50%)	7 (64%)
Total	206	143 (69%)	96 (67%)
**Gender**			
Female	185	127	83
Male	21	16	13
**Place of residence**			
*Australasia*			
Australia	105	66	45
New Zealand	3	3	1
Fiji	1	1	0
*Europe*			
United Kingdom	23	21	11
Ireland	10	4	2
Netherlands	5	5	3
Sweden	5	5	5
Norway	4	3	2
Bulgaria	1	0	0
France	1	1	1
Georgia	1	1	1
Macedonia	1	1	1
Moldova	1	0	0
Poland	1	1	1
*Asia*			
Israel	4	2	1
Hong Kong	1	1	1
Indonesia	1	0	0
Kazakhstan	1	1	0
Malaysia	1	1	1
Singapore	1	1	1
*North America*			
United States of America	28	22	16
Canada	2	0	0
Guatemala	1	0	0
*South America*			
Brazil	1	0	0
*Africa*			
South Africa	3	3	3

*R1* = Round 1, *R2* = Round 2, *R3* = Round 3.

An additional 78 outcomes were suggested by round 1 participants. Additional proposed outcomes were reviewed and after consultation with the Steering Group, an additional seven outcomes that were deemed substantively different to already included outcomes were included in subsequent rounds (Supplementary Table S3).

Round 2 had 143 participants (69% of Round 1 participants). Round 2 participants re-rated all round 1 outcomes and also rated the seven additional outcomes suggested in round 1 (Supplementary Table S3). In round 2, six outcomes reached consensus for inclusion in the COS: the five outcomes that had reached consensus in round 1 (‘child time spent sedentary’, ‘child physical activity’, ‘child diet quality’, ‘child dietary intake’, ‘household food security’) plus ‘parent/caregiver role modelling of healthy eating’ (Supplementary Table S4). No outcomes met the consensus criteria to exclude as a core outcome, and so all outcomes were again brought forward to round 3 of the e-Delphi survey.

In round 3, 96 participants re-rated 119 outcomes (47% of round 1 participants; 67% of round 2 participants). In round 3, ten outcomes reached consensus for inclusion in the COS: the six outcomes that had reached consensus in round 2 (‘child time spent sedentary’, ‘child physical activity’, ‘child diet quality’, ‘child dietary intake’, ‘household food security’, ‘parent/caregiver role modelling of healthy eating’) plus ‘child screen time’, ‘sedentary behaviour or physical activity home environment’, ‘child non-core beverages intake’ and ‘food environment’(Supplementary Tables S4, S5). Analysis of the distribution of scores across all outcomes, comparing between the scores of participants who only completed round 1 versus participants who completed all rounds suggests that participants who completed the study scored the outcomes similarly to those who withdrew or dropped out from the study (Supplementary Fig. S1). No outcomes met the consensus criteria to exclude as a core outcome. All outcomes were therefore carried forward to the consensus meeting.

### Consensus meeting

Nine stakeholders participated in the online consensus meeting: two policy-makers, two researchers, two parents/caregivers, one health professional and two community or organisational stakeholders. Participants were from Australia (*n* = 8) and the South Pacific (*n* = 1). Eight participants were female, with one male participant. After presentation and discussion of the outcomes, 22 were voted as critical for inclusion in the COS (Table 3). Following discussion by all participants and group consensus, ‘parent/caregiver role modelling of healthy eating’ was reworded as a subset of ‘parent/caregiver nutrition parenting practices’.

**Table 3:** Final core outcome set outcomes, by outcome domain.

Outcome	Outcome domain	Outcome definition
Child weight-based anthropometry	Anthropometry	Includes child height, weight, body mass index (BMI), BMI z-score, percentile, prevalence of overweight and obesity, weight for height or length.
Child diet quality	Dietary intake* (in children aged from 1 to 5 years)	Includes child’s diet quality in relation to the proportion of less healthy foods in relation to overall energy intake, the proportion of healthy foods in relation to overall energy intake, the intake of nutrients compared to dietary guideline recommendations, the intake of a healthy dietary pattern, the change in healthy meals consumed.
Child dietary intake		Includes quantity or frequency of child’s general dietary intake.
Child fruit and vegetable intake		Includes quantity or frequency of child’s consumption of fruits and/or vegetables.
Child non-core food intake		Includes quantity or frequency of child’s consumption of processed snack foods, energy dense sweets (e.g., chocolate bars, ice cream), high caloric foods, savoury snacks, discretionary calories, french fries, fast or deep-fried foods.
Child non-core beverages intake		Includes quantity or frequency of consumption of sodas, soft drinks, sugar-sweetened beverages, juices, cordials, high caloric drinks by the child.
Child meal patterns		Includes child meal times and snack patterns, including the number and/or timing of meals per day.
Child screen time	Sedentary behaviour	Includes time spent by the child watching DVDs, TV, videos or using computers, tablets, smart phones or other portable electronic devices.
Child time spent sedentary		Includes time spent by the child sitting, lying awake, immobile but awake, or with no trunk movement. Includes time spent in restricted movement (e.g., in a car seat or a stroller). Does not include time asleep or screen time.
Child physical activity	Physical activity	Includes frequency or time spent by the child in physical activity, including total physical activity, moderate-to-vigorous physical activity, leisure physical activity, walking, crawling. Includes organised physical activity (e.g., frequency or time spent by the child at sport, dance, swimming) and unorganised physical activity (e.g., frequency of time spent by the child in energetic play, play, active play, running, jumping, climbing, structured free play or common play activities, frequency or time spent by the child at the park, playground, excursions).
Infant tummy time		Includes commencement age, frequency and/or time spent by the infant in tummy time. Specifically for trials involving infants.
Child sleep duration	Sleep	Includes child sleep quantity, hours of total daily sleep duration, total minutes of sleep in 24-hour period, the average length of a sleep bout and duration of individual sleep bouts, average night-time sleep, sleep hours per night, sleep consecutive hours at night, rate of sleeping through the night.
Parent/caregiver physical activity parenting practices	Outcomes in parents/caregivers	Includes parent/caregiver rules and practices, intentions or habit strength for activity behaviours, co-participation and role-modelling.
Parent/caregiver sleep parenting practices		Includes parent/caregiver rules and practices for sleep, bedtime or sleep routine, assistance to sleep by touching, intervening when awake in the night.
Parent/caregiver nutrition parenting practices		Includes parent/caregiver rules and practices for nutrition and/or feeding, trying new foods, intentions or habit strength for nutrition behaviours, role-modelling.
Food environment	Environmental	Includes environments in which food is consumed, available, purchased, available to be purchased. Includes proximity to food store locations, the distribution of food store locations, accessibility and convenience of food sources.
Household food security		Reliable access to healthy and nutritious foods.
Family meal environment		Includes features of the environment where meals are eaten. Includes frequency of eating meals together as a family, media or device use at family meals, eating at dining table or similar.
Sedentary behaviour or physical activity home environment		Includes features of the home environment that may influence sedentary behaviour or physical activity, including fixed and non-fixed home and yard features. For example, size of backyard, presence of playground or play equipment.
Early childhood education and care (ECEC) environment		Includes fixed or non-fixed environmental features of home or centre-based care, preschool or kindergarten to address obesity-related behaviours. Fixed features might include playgrounds, kitchens. Non-fixed features might include service organisation and policy environment.
Child wellbeing	Emotional or cognitive functioning	Includes social-emotional wellbeing and emotional health of the child.
Economic evaluation	Economic	Includes any form of economic evaluation, including cost-effectiveness analysis, cost-utility analysis.

*The COS for infant feeding interventions [8] should be consulted for interventions in children aged ≤ 1 year of age.

## Discussion

The finalised COS-EPOCH includes 22 outcomes that should be measured and reported in trials of early childhood obesity prevention interventions commencing in children aged from birth to 5 years. The six core outcomes related to dietary intake complement the outcomes included in an existing COS for early infant feeding interventions in the first year of life, that has already been published [8]. The COS-EPOCH provides additional guidance to researchers and trialists on the dietary intake domain outcomes for collection and reporting in trials in children aged from 1 up to 5 years. The COS-EPOCH also includes a further 16 outcomes related to eight outcome domains (anthropometry, sedentary behaviour, physical activity, sleep, outcomes in parents/caregivers, environmental, emotional or cognitive functioning, economic). This means COS now cover all key domains relevant to obesity prevention, as identified by relevant international stakeholders, and facilitates consistent and comprehensive assessment of outcomes to enable comparison between early childhood obesity prevention interventions.

It is well-recognised that the determinants and drivers of childhood obesity are complex, and that interventions must address different layers of the ecological model (e.g., child, caregiver, environment, community/policy) to influence health and wellbeing [29]. This is reflected in the COS-EPOCH, with the inclusion of outcomes related to energy balance behaviours at the individual level (e.g., physical activity, sleep duration, dietary intake), outcomes in parents/caregivers reflecting the family/social level (e.g., parent/caregiver nutrition, physical activity and sleep parenting practices), and environmental and policy-relevant outcomes (e.g., early childhood education and care (ECEC) environment, economic evaluation). While the COS-EPOCH includes 22 outcomes for collection and reporting, some trialist discretion is recommended to tailor its application to specific needs. For instance, while the COS-EPOCH recommends the collection and reporting of infant tummy time this may not be relevant for an intervention beginning at child age of 4 years. Similarly, the collection and reporting of outcomes from all domains may be deemed less appropriate to an intervention that focuses only on a subset of energy balance behaviours. In addition, for some trials the collection and reporting of 22 outcomes may not be feasible given study resourcing limitations. In these instances trialists should at a minimum be able to justify why they have not collected and reported outcomes across all COS-EPOCH domains, and why they have not measured certain outcomes.

This study followed published best-practice guidelines for the development and reporting of COS [7, 13]. Strengths of the study included concordance with a published study protocol [11], and participation of international participants in the e-Delphi and consensus meeting. While a large number of countries were represented in the e-Delphi, it should be noted that the majority of participants were from Australia, followed by the United Kingdom and United States of America. The use of a professional facilitator for the consensus meeting avoided researcher bias or undue influence on the finalised COS, and ensured that all stakeholders could freely express their views during COS development [7]. The Steering Group providing expert oversight and guiding the development of the COS comprised experts in the field of early childhood obesity prevention interventions. The Steering Group was at all times guided by published guidance on COS development [7] and did not participate in either the Delphi study or consensus meeting, minimising researcher bias or undue influence on the finalised COS. The publication of a pre-defined study protocol [11], which outlined the research design, the decision points and the decision rules used to develop the COS also sought to minimise any undue influence of the Steering Group. While a limitation of this study is the absence of a non-academic stakeholder member to the Steering Group, the voices of non-academics and end users/beneficiaries (e.g. policy-makers, parents) were represented in the project findings as study participants. The timeline of the study meant that participant recruitment and engagement with the e-Delphi study and the consensus meeting occurred during periods of COVID19 pandemic-related disruption and so the use of virtual technologies was a significant strength of the study. Perhaps due to this pandemic-related disruption, e-Delphi participation from some stakeholder groups, and in particular parents/caregivers, was lower than expected. Two parents/caregivers were however able to participate in the virtual consensus meeting, and the use of the experienced facilitator ensured that their voices were able to be heard in the consensus process [7]. While the use of technologies allowed for international participation in COS development, the virtual consensus meeting was conducted in Australia and this likely restricted participation due to time zone differences for many potential participants located in other hemispheres. The Australian-centric nature of the virtual consensus meeting is also a limitation of this study. Finally, while e-Delphi and consensus participants came from a total of 25 countries, more investigation into the utility of the COS in LMIC, or non-English speaking countries, in particular is warranted given the skew towards participants from western countries in the development of the COS [30].

The COS-EPOCH represents the first COS for early childhood obesity interventions inclusive of a wide range of risk factors and conducted across a wide range of settings. Our scoping review identified a large number of outcomes that are currently being collected and reported in early childhood obesity prevention interventions, supporting the need for a standardised set of core outcomes in this area. Through the development and use of the COS-EPOCH, it is hoped that more standardised data collection and reporting can lead to more efficient intervention evaluation and knowledge synthesis to inform future intervention development and research translation efforts [8]. A recent systematic review identified barriers to the uptake of COS by trialists, and found lack of awareness of existence of the COS and lack of validated instrument measures were key barriers [31]. Informed by these findings dissemination of the COS-EPOCH will aim to maximise awareness and use of the COS. Dissemination approaches will include presentations at relevant conferences and seminars, and an infographic has been developed for dissemination to relevant stakeholders, including government, academic, community and organisational stakeholders interested in early childhood obesity prevention interventions. A program of work is also underway to identify outcome measurement instruments to recommend, to facilitate the collection of core outcomes in the COS-EPOCH following the COSMIN guidelines [32, 33].

## Conclusions

The Core Outcome Set for Early intervention trials to Prevent Obesity in Childhood (COS-EPOCH) followed a rigorous and validated approach that identified 22 outcomes across nine outcome domains for collection and reporting in all early obesity prevention intervention studies. Future research will identify recommended outcome measurement instruments for each of the outcomes included in the COS-EPOCH, to further facilitate and consolidate outcome selection and reporting. Development and uptake of the COS-EPOCH will assist in more streamlined syntheses of findings across research studies, potentially making better use of the learnings from early childhood obesity prevention intervention studies. A more consistent approach to measurement and reporting of this minimum set of outcomes that is considered essential across stakeholder groups will lead to more timely, robust and comprehensive evaluation of the effects of early childhood obesity prevention interventions, and will better support a comprehensive understanding of the effects of interventions and the mechanisms for those effects.

## Supplementary information

Supplementary File

## Data Availability

The datasets generated during and/or analysed during the current study are available from the corresponding author on reasonable request.
