# Supplementary material for: Core outcome set for early intervention trials to prevent obesity in childhood (COS-EPOCH): Agreement on “what” to measure
Source: Int J Obes (Lond). 2022 Aug 4;46(10):1867–74. doi: 10.1038/s41366-022-01198-w (PMC9492532; doi:10.1038/s41366-022-01198-w)
Supplement: Supplementary file 1 — Supplementary File [file 41366_2022_1198_MOESM1_ESM.pdf]

Core Outcome Sets for Early intervention trials to Prevent Obesity in CHildhood (COS-EPOCH): Agreement on "what" to measure

Vicki Brown [1,2], Marj Moodie [1,2], Marufa Sultana [1], Kylie E Hunter [2,3], Rebecca Byrne [2,4], Anna Lene Seidler [2,3], Rebecca Golley [2,5], Rachael W Taylor [2,6], Kylie D Hesketh [2,7], Karen Matvienko-Sikar [8]

1 Deakin University, Geelong, Deakin Health Economics, Institute for Health Transformation, Global Obesity Centre (GLOBE), School of Health and Social Development, Victoria 3220, Australia

2 Centre for Research Excellence in the Early Prevention of Obesity in Childhood, University of Sydney, Sydney NSW, Australia

3 NHMRC Clinical Trials Centre, University of Sydney, Sydney NSW, Australia

4 Queensland University of Technology, School of Exercise and Nutrition Sciences

5 Caring Futures Institute, College of Nursing and Health Sciences, Flinders University, SA 5042, Australia

6 Department of Medicine, University of Otago, Dunedin, New Zealand

7 Deakin University, Geelong Victoria 3220, Institute for Physical Activity and Nutrition

8 School of Public Health, University College Cork, Ireland

Corresponding author: Dr Vicki Brown, Deakin University, Geelong, Deakin Health Economics, Institute for Health Transformation, Global Obesity Centre (GLOBE), School of Health and Social Development, Victoria 3220, Australia. victoria.brown@deakin.edu.au

List of Tables

Supplementary Table S1- Core Outcome Set-STAndards for Reporting (1) 3

Supplementary Table S2- Recommendations for the Conducting and Reporting of Delphi Studies (CREDES)(2) 5

Supplementary Table S3- Outcomes included in the e-Delphi survey, by round 7

Supplementary Table S4- Summary results by stakeholder group and eDelphi round 18

Supplementary Table S5 - Percentage of stakeholders who scored the outcome as critical (score of 7–9) for inclusion, Round 3 97

List of Figures

Supplementary Figure S1 – Example of graphical feedback given to participants between rounds . 102

Supplementary Figure S2 - Distribution of scores across all outcomes 103

Supplementary Table S1- Core Outcome Set-STAndards for Reporting (1)

**Section/topic**	**Item No.**	**Checklist item**	**Manuscript page**
**Title/abstract**			
Title	1a	Identify in the title that the paper reports the development of a COS.	1,3
Abstract	1b	Provide a structured summary.	1
**Introduction**			
Background and objectives	2a	Describe the background and explain the rationale for developing the COS.	3
	2b	Describe the specific objectives with reference to developing a COS.	4
Scope	3a	Describe the health condition(s) and population(s) covered by the COS.	4
	3b	Describe the intervention(s) covered by the COS.	4
	3c	Describe the setting(s) in which the COS is to be applied.	4
**Methods**			
Protocol/Registry entry	4	Indicate where the COS development protocol can be accessed, if available, and/or the study registration details.	4
Participants	5	Describe the rationale for stakeholder groups involved in the COS development process, eligibility criteria for participants from each group, and a description of how the individuals involved were identified.	5
Information sources	6a	Describe the information sources used to identify an initial list of outcomes.	5
	6b	Describe how outcomes were dropped/combined, with reasons (if applicable).	6
Consensus process	7	Describe how the consensus process was undertaken.	6
Outcome scoring	8	Describe how outcomes were scored and how scores were summarised.	8
Consensus definition	9a	Describe the consensus definition.	9
	9b	Describe the procedure for determining how outcomes were included or excluded from consideration during the consensus process.	9, 10

Ethics and consent	10	Provide a statement regarding the ethics and consent issues for the study.	5
**Results**			
Protocol deviations	11	Describe any changes from the protocol (if applicable), with reasons, and describe what impact these changes have on the results.	n/a
Participants	12	Present data on the number and relevant characteristics of the people involved at all stages of COS development.	11, 12, 13
Outcomes	13a	List all outcomes considered at the start of the consensus process.	Supp Table S3
	13b	Describe any new outcomes introduced and any outcomes dropped, with reasons, during the consensus process.	12, Supp Table S3
COS	14	List the outcomes in the final COS.	13
**Discussion**			
Limitations	15	Discuss any limitations in the COS development process.	15
Conclusions	16	Provide an interpretation of the final COS in the context of other evidence, and implications for future research.	14,15,16
Other information			
Funding	17	Describe sources of funding/role of funders.	18
Conflicts of interest	18	Describe any conflicts of interest within the study team and how these were managed.	18

Supplementary Table S2- Recommendations for the Conducting and Reporting of Delphi Studies (CREDES)(2)

**Item no.**	**Recommendation**	**Manuscript page**
Rationale for the choice of the Delphi technique		
1	Justification. The choice of the Delphi technique as a method of systematically collating expert consultation and building consensus needs to be well justified. When selecting the method to answer a particular research question, it is important to keep in mind its constructivist nature	4, 6
Planning and design		
2	Planning and process. The Delphi technique is a flexible method and can be adjusted to the respective research aims and purposes. Any modifications should be justified by a rationale and be applied systematically and rigorously.	6-9
3	Definition of consensus. Unless not reasonable due to the explorative nature of the study, an a priori criterion for consensus should be defined. This includes a clear and transparent guide for action on (a) how to proceed with certain items or topics in the next survey round, (b) the required threshold to terminate the Delphi process and (c) procedures to be followed when consensus is (not) reached after one or more iterations.	9
Study conduct		
4	Informational input. All material provided to the expert panel at the outset of the project and throughout the Delphi process should be carefully reviewed and piloted in advance in order to examine the effect on experts' judgements and to prevent bias.	6-9
5	Prevention of bias. Researchers need to take measures to avoid directly or indirectly influencing the experts' judgements. If one or more members of the research team have a conflict of interest, entrusting an independent researcher with the main coordination of the Delphi study is advisable.	6-9
6	Interpretation and processing of results. Consensus does not necessarily imply the 'correct' answer or judgement; (non)consensus and stable disagreement provide informative insights and highlight differences in perspectives concerning the topic in question.	6-9
7	External validation. It is recommended to have the final draft of the resulting guidance on best practice reviewed and approved by an external board or authority before publication and dissemination.	5, 6-9
Reporting		
8	Purpose and rationale. The purpose of the study should be clearly defined and demonstrate the appropriateness of the use of the Delphi technique as a method to achieve the research aim. A rationale for the choice of the Delphi technique as the most suitable method needs to be provided.	4, 6-9

9	Expert panel. Criteria for the selection of experts and transparent information on recruitment of the expert panel, sociodemographic details including information on expertise regarding the topic in question, (non)response and response rates over the ongoing iterations should be reported	6-9, 11-13, Table 2
10	Description of the methods. The methods employed need to be comprehensible; this includes information on preparatory steps (How was available evidence on the topic in question synthesised?), piloting of material and survey instruments, design of the survey instrument(s), the number and design of survey rounds, methods of data analysis, processing and synthesis of experts' responses to inform the subsequent survey round and methodological decisions taken by the research team throughout the process.	6-9
11	Procedure. Flow chart to illustrate the stages of the Delphi process, including a preparatory phase, the actual 'Delphi rounds', interim steps of data processing and analysis, and concluding steps.	11, Figure 1
12	Definition and attainment of consensus. It needs to be comprehensible to the reader how consensus was achieved throughout the process, including strategies to deal with non-consensus.	Table 1
13	Results. Reporting of results for each round separately is highly advisable in order to make the evolving of consensus over the rounds transparent. This includes figures showing the average group response, changes between rounds, as well as any modifications of the survey instrument such as deletion, addition or modification of survey items based on previous rounds.	11-13, Supp Table S4
14	Discussion of limitations. Reporting should include a critical reflection of potential limitations and their impact of the resulting guidance.	15
15	Adequacy of conclusions. The conclusions should adequately reflect the outcomes of the Delphi study with a view to the scope and applicability of the resulting practice guidance.	14-17
16	Publication and dissemination. The resulting guidance on good practice in palliative care should be clearly identifiable from the publication, including recommendations for transfer into practice and implementation. If the publication does not allow for a detailed presentation of either the resulting practice guidance or the methodological features of the applied Delphi technique, or both, reference to a more detailed presentation elsewhere should be made (e.g. availability of the full guideline from the authors or online; publication of a separate paper reporting on methodological details and particularities of the process (e.g. persistent disagreement and controversy on certain issues)). A dissemination plan should include endorsement of the guidance by professional associations and health care authorities to facilitate implementation.	14-17

Supplementary Table S3- Outcomes included in the e-Delphi survey, by round

Outcome domain	Outcome	Definition	Round/s
Anthropometry	Child weight-based anthropometry	Includes child height, weight, body mass index (BMI), BMI z-score, percentile, prevalence of overweight and obesity, weight for height or length.	1,2,3
	Child body composition	Proportion of fat, muscle, bone and water. Includes child lean body mass, body fat, percent body fat, fat free mass, abdominal fat, skinfolds.	1,2,3
	Child circumference	Includes child waist circumference, waist to height/length, arm circumference, head shape or circumference.	1,2,3
Sedentary behaviour	Child screen time	Includes time spent by the child watching DVDs, TV, videos or using computers, tablets, smart phones or other portable electronic devices.	1,2,3
	Sedentary behaviour or physical activity home environment	Includes measures of the home environment that may influence sedentary behaviour or physical activity, including fixed and non-fixed home and yard features. For example, size of backyard, presence of playground or play equipment.	1,2,3
	Child time spent sedentary	Includes time spent by the child sitting, lying awake, immobile but awake, or with no trunk movement. Includes time spent in restricted movement (e.g. in a carseat or a stroller). Does not include time asleep or screen time.	1,2,3
Physical activity	Child active transport	Includes frequency, duration, use of active transport by the child (e.g. walking, cycling, public transport or scooting).	1,2,3
	Child physical fitness	Includes measures of child physical fitness, including shuttle run tests, handgrip strength tests, standing long jump tests, one-leg stance tests.	1,2,3
	Child active indoor play	Includes frequency or time spent by the child in active indoor play, playing indoors, indoor play in a playful context combined with activity that is above resting metabolic rate.	1,2,3

	Child active outdoor play	Includes frequency or time spent outdoors by the child, playing outdoors, in active outdoor play or outdoor play in a playful context combined with activity that is above resting metabolic rate.	1,2,3
	Child physical activity	Includes frequency or time spent by the child in physical activity, including total physical activity, moderate-to-vigorous physical activity, leisure physical activity, walking, crawling. Includes organised physical activity (e.g. frequency or time spent by the child at sport, dance, swimming) and unorganised physical activity (e.g. frequency of time spent by the child in energetic play, play, active play, running, jumping, climbing, structured free play or common play activities, frequency or time spent by the child at the park, playground, excursions).	1,2,3
	Infant tummy time	Includes commencement age, frequency, time spent by the infant in tummy time.	1,2,3
	Infant floor based play time	Includes time spent by the infant playing on the floor, unconfined or unrestrained.	1,2,3
	Child enjoyment of physical activity and playing	Includes child's enjoyment of playing with parents, child's enjoyment of physical activity (movement, play), child’s enjoyment of playing with objects and toys.	1,2,3
Sleep	Child sleep duration	Includes child sleep quantity, hours of total daily sleep duration, total minutes of sleep in 24-hour period, the average length of a sleep bout and duration of individual sleep bouts, average night time sleep, sleep hours per night, sleep consecutive hours at night, rate of sleeping through the night. Can be used to derive child awake time.	1,2,3
	Child sleep disturbance or problems	Includes difficulty sleeping, difficulty falling asleep in own bed, sleep reluctance, disturbances in the amount or quality of sleep, night wakings of the child.	1,2,3
	Child sleep efficiency	The percentage of total time the child spends in bed actually spent in sleep.	1,2,3
	Child sleep latency	The time it takes the child to fall asleep.	1,2,3

	Child sleep patterns or timing	Includes napping or sleep patterns, timing of sleeps of the child.	1,2,3
	Child sleep quality	Quality of sleep of the child.	1,2,3
Dietary intake	Child carbohydrate intake or glycemic load	Includes quantity or frequency of carbohydrate intake by the child, glycemic load of the child (rates carbohydrates according to how quickly they raise blood glucose).	1,2,3
	Child dairy intake	Includes quantity or frequency of dairy intake by the child. Also includes child's requests for calcium, dairy or milk products.	1,2,3
	Child diet quality	Includes child's diet quality in relation to the proportion of less healthy foods in relation to overall energy intake, the proportion of healthy foods in relation to overall energy intake, the intake of nutrients compared to dietary guideline recommendations, the intake of a healthy dietary pattern, the change in healthy meals consumed; child healthy eating index score or child nutrition risk scores.	1,2,3
	Child dietary intake	Includes quantity or frequency of child's general dietary intake.	1,2,3
	Child fat intake	Includes quantity or frequency of child's fat intake.	1,2,3
	Child fibre intake	Includes quantity or frequency of child's fibre intake.	1,2,3
	Child fruit and vegetable intake	Includes quantity or frequency of child's consumption of fruits and/or vegetables.	1,2,3
	Child grains, breads, cereals intake	Includes quantity or frequency of child's consumption of cereals, bread, pasta, rice, porridge, whole grains.	1,2,3
	Child meat, poultry, fish intake	Includes quantity or frequency of child's consumption of meat, poultry, fish.	1,2,3
	Child non-core food intake	Includes quantity or frequency of child's consumption of processed snack foods, energy dense sweets (e.g. chocolate bars, ice cream), high caloric foods, savoury snacks, discretionary calories, french fries, fast or deep-fried foods.	1,2,3

	Child non-core beverages intake	Includes quantity or frequency of consumption of sodas, soft drinks, sugar-sweetened beverages, juices, cordials, high caloric drinks by the child.	1,2,3
	Child out of home foods intake	Includes quantity or frequency of child's consumption of meals not prepared in the home, commercially prepared meals, fast foods, eating in restaurants.	1,2,3
	Child protein intake	Includes quantity or frequency of child's protein intake.	1,2,3
	Child sodium intake	Includes quantity or frequency of child's sodium intake.	1,2,3
	Child total and added sugars intake	Includes quantity or frequency of child's consumption of total and added sugars, foods with added sugars.	1,2,3
	Child desire for drinks	Includes child's desire or requests for drinks.	1,2,3
	Child eating in response to emotion	Child eating more or less, in response to emotional state, emotional undereating, emotional overeating.	1,2,3
	Child enjoyment of food	Includes child's response to the food, enjoyment of food.	1,2,3
	Child meal patterns	Includes child meal times and snack patterns, number of meals per day.	1,2,3
	Child food neophobia	Includes child reluctance to eat, selectivity about the range of foods accepted or the avoidance or unwillingness to eat new foods.	1,2,3
	Child satiety responsiveness	Includes child feeding self-regulation, stopping eating when full.	1,2,3
	Child slowness in eating	Child dawdling over meals, eating slowly.	1,2,3
Outcomes in parents/caregivers	Parent/caregiver weight based anthropometry	Includes parent/caregiver height, weight, body mass index (BMI), BMI z-score, percentile, prevalence of overweight and obesity, weight for height or length.	1,2,3
	Parent/caregiver screen time parenting practice	Includes rules around screen time in the home or early education and care settings. Includes parents' use of TV viewing as a form of family recreation,	1,2,3

		frequency of TV viewing during mealtimes, presence and availability of TV and media devices, total time TV is on when no one is watching.	
	Parent/caregiver role modelling of sedentary behaviours	Includes frequency, time spent by the parent/caregiver in sedentary activities, such as watching TV, sitting, lying still.	1,2,3
	Parent/caregiver role modelling of physical activity	Includes frequency or time spent by the parent/caregiver in physical activity, or work-related physical activities.	1,2,3
	Parent/caregiver role modelling of healthy eating	Parent/caregiver role modelling of healthy eating (e.g. eating a wide variety of fruits and vegetables, minimising discretionary food intake)	1,2,3
	Parent/caregiver encouragement of physical activity	Includes encouragement of physical activity by parent/caregiver, support for physical activity.	1,2,3
	Parent/caregiver co-participation in physical activity	Includes engagement of parent/caregiver in physical activity or physically active play with child.	1,2,3
	Parent/caregiver physical activity parenting practice	Includes parent/caregiver rules and practices, intentions or habit strength for activity behaviours.	1,2,3
	Parent/caregiver sleep parenting practice	Includes general sleep parent/caregiver practices, bedtime or sleep routine, assistance to sleep by touching, intervening when awake in the night.	1,2,3
	Parent/caregiver daytime dysfunction or fatigue	Parent/caregiver difficulty in carrying out tasks due to tiredness or sleepiness. Parent/caregiver feeling of extreme or constant tiredness.	1,2,3
	Parent/caregiver nutrition parenting practices	Includes parent/caregiver rules about nutrition, trying new foods, habit strength for new feeding behaviours.	1,2,3
	Parent/caregiver's dietary intentions	Includes parent/caregiver's readiness or intention to eat more fruit and vegetables, habit strength for dietary behaviours.	1,2,3
	Parent/caregiver food variety	Includes variation in types of foods offered by the parent/caregiver.	1,2,3

	Parent/caregiver anxiety symptoms	Parent/caregiver symptoms of anxiety.	1,2,3
	Parent/caregiver concerns about child's weight	Parent/caregiver concerns about child's weight.	1,2,3
	Parent/caregiver depression symptoms	Parent/caregiver symptoms of depression.	1,2,3
	Parent/caregiver self-efficacy	Includes parent/caregiver self-efficiency, self-efficacy, confidence, self-agency for promoting healthy behaviours.	1,2,3
	Parent/caregiver social support	Includes parent/caregiver support, partner and family support.	1,2,3
	Parent/caregiver stress	Parent/caregiver feeling of emotional tension.	1,2,3
	Parent/caregiver attitudes	Parent/caregiver attitudes towards obesogenic behaviours, a healthy lifestyle, nutrition, physical activity, feeding.	1,2,3
	Parent/caregiver awareness of behaviours	Parent/caregiver awareness of physical activity behaviours and feeding practices.	1,2,3
	Parent/caregiver beliefs	Parent/caregiver beliefs in relation to feeding, healthy eating, physical activity, feeding.	1,2,3
	Parent/caregiver health literacy	The skills and knowledge of a person to access, understand and use information to make decisions, and take action about health and healthcare, food, physical activity.	1,2,3
	Parent/caregiver knowledge	Parent/caregiver knowledge of food, feeding, nutrition, physical activity, exercise, healthy eating, active play, sedentary behaviours, the sustainability consequences of food and toy selections, healthy lifestyles.	1,2,3
	Parent/caregiver readiness to change	Parent/caregiver motivation, readiness for lifestyle change.	1,2,3

	Parenting style	Includes parental style in relation to general parenting, healthy behaviours, discipline, rules, anxiety, nurturance, involvement, routine, anxiety, parental authority, cry tolerance and limit setting abilities.	1,2,3
	Parent/caregiver responsiveness	Defined as the sensitive and accepting behaviours identified in a parent/caregiver toward a child.	1,2,3
	Family functioning	The social and structural properties of the global family environment.	1,2,3
	Parent/caregiver perception of child weight	Parent/caregiver perception of child weight, weight status, BMI, obesity risk.	1,2,3
	Perception of parent/caregiver influence on child's health behaviours	Perception of parent/caregiver influence on child's health behaviours.	1,2,3
	Parent/caregiver perceptions of adequacy of resources and barriers	Includes parent/caregiver perceptions of time and resource barriers to meal preparation, adequacy of resources needed to plan and prepare meals.	1,2,3
Feeding	Parent/caregiver feeding control	Includes parent/caregiver feeding control practices, restriction, covert restriction, pressure to eat, monitoring.	1,2,3
	Parent/caregiver emotional feeding	Includes parent/caregiver using food to regulate the child’s emotional states.	1,2,3
	Parent/caregiver feeding encouragement	Includes parent/caregiver encouraging child’s involvement in meal planning and preparation, promoting well-balanced food intake, teaching about nutrition.	1,2,3
	Parent/caregiver feeding interaction	Includes parent/caregiver child-feeding interaction, encouragement to eat.	1,2,3
	Parent/caregiver feeding structure	Includes positive, structure based feeding practices (limit-setting, consistent routines) that promote self-regulation.	1,2,3

	Parent/caregiver feeding style	Parent/caregiver style in relation to feeding.	1,2,3
	Parent/caregiver using food as a reward	Includes parent/caregiver using food as a reward.	1,2,3
	Parent/caregiver frequency of bottle use	Frequency of bottle use by parent/caregiver to feed child.	1,2,3
	Parent/caregiver promoting autonomy	Includes parent/caregiver promoting internal control of eating, parents/caregivers allow the child control of his/her eating behaviours.	1,2,3
	Parent/caregiver that is responsible for feeding	Includes person/s responsible for feeding.	1,2,3
Environmental	Food environment	Includes environments in which food is consumed, available, purchased, available to be purchased. Includes proximity to food store locations, the distribution of food store locations, accessibility and convenience of food sources.	1,2,3
	Household food security	Reliable access to healthy and nutritious foods.	1,2,3
	Family meal environment	Includes frequency of eating meals together as a family with the TV off, number of days that the child had dinner while watching TV, media device use at family meals, no. of days when healthy mealtimes occurred, family meals in car, at fast food restaurant, at dining table, eating alone.	1,2,3
	Early childhood education and care (ECEC) environment	Includes fixed or non-fixed environmental features of childcare, preschool or kindergarten to address obesity-related behaviours (e.g. poor nutrition or diet, insufficient physical activity, excess sedentary behaviour, insufficient sleep). Fixed features might include playgrounds, kitchens. Non-fixed features might include service organisational and policies, equipment.	1,2,3
	Healthcare environment	Physical environment in which healthcare is accessed.	1,2,3

Emotional or cognitive functioning	Child internalising and/or externalising behaviours	Aggressive, internalising and externalizing behaviours in child.	1,2,3
	Child emotion regulation	Includes reports of child anger, frustration, temperament, emotional coping, emotion self-regulation.	1,2,3
	Child emotional development	The ability of the child to recognise, express, and manage feelings and to have empathy for the feelings of others.	1,2,3
	Child school readiness	A measure of the knowledge, skills and behaviours that enable children to participate and succeed in school.	1,2,3
	Child wellbeing	Includes social-emotional wellbeing and emotional health of the child.	1,2,3
	Child attention control	Child's ability to stay focused.	1,2,3
	Child cognitive development	Includes child communication, problem solving and personal social skills.	1,2,3
	Child executive function	Includes tests for child's working memory, inhibition.	1,2,3
	Child self-regulation	Includes child's ability to stop doing a task, inhibitory control, self-regulation.	1,2,3
Motor skill development	Child fundamental movement skills	Includes motor skills, locomotor skills (run, gallop, hop, leap, horizontal jump, slide and balance), object control skills (striking a stationary ball, stationary dribble, catch, kick, overhand throw and underhand roll, shuttle run (speed), standing long jump (speed strength), one leg stand (coordination, static balance), sit and reach (flexibility), and lateral jumping (coordination, strength endurance), motor agility, dynamic balance.	1,2,3
Perceptions of child/parent/caregiver/family and preferences	Child activity preference or perception	Includes change in liking, degree of liking activity by child.	1,2,3
	Child food preference	Includes change in liking, degree of liking food by child.	1,2,3

Blood and lymphatic system	Child blood pressure	Blood pressure of the child.	1,2,3
	Child cholesterol	Cholesterol of the child.	1,2,3
	Child glucose	Glucose of the child.	1,2,3
	Child hemoglobin	Hemoglobin of the child.	1,2,3
	Child lipids	Lipids of the child.	1,2,3
	Child metabolic and inflammatory parameters	Metabolic and inflammatory parameters of the child.	1,2,3
Quality of life	Child health-related quality of life	An individual's or a group's perceived physical and mental health over time.	1,2,3
Economic	Economic evaluation	Includes any form of economic evaluation, including cost-effectiveness analysis, cost-utility analysis.	1,2,3
	Child healthcare utilisation	Includes cost and quantity of health care or services used by the child.	1,2,3
	Intervention cost	Cost of intervention.	1,2,3
Oral health	Child oral hygiene	Includes tooth-brushing and oral and dental behaviours of the child.	1,2,3
	Child dental caries	Includes presence, number, rate and severity of caries in the child.	1,2,3
Anthropometry	Birth weight adjusted for gestational age	Child weight at birth adjusted for gestational age	2,3
Environmental	Household Chaos	Household chaos is characterized by high levels of confusion, disorganisation and hurriedness in the home	2,3
Outcomes in parents/caregivers	Parent/caregiver body image and satisfaction	Parent/caregiver thoughts and feelings about their body, including satisfaction or dissatisfaction about shape, weight and body parts.	2,3

Emotional or cognitive functioning	Child self esteem	Child liking themself, believing in themself, feeling worthwhile and knowing what they do well.	2,3
	Parent child mutually responsive orientation	Mutually responsive orientation encompasses shared cooperation and shared positive affect between parent and child.	2,3
Dietary intake	Child fruit intake	Includes quantity or frequency of child's consumption of fruits (separate from vegetable intake).	2,3
	Child vegetable intake	Includes quantity or frequency of child's consumption of vegetables (separate from fruit intake).	2,3

Supplementary Table S4- Summary results by stakeholder group and eDelphi round

	**Health professionals**	**Community or organisational**	**Parent/caregiver**	**Policy-maker or funder**	**Researcher**	**Mean of stakeholder group mean scores**
**01 - Child weight based anthropometry**						
**Round 1**						
Mean score	7	6.7	6.5	**8.0**	**8.1**	7.3
Median score	8	7	7	**9**	**9**	
MADM	1.4	0.9	1.3	**1.1**	**0.9**	
Agreement	Low agreement	High agreement	High agreement	**High agreement**	**High agreement**	
	No consensus	No consensus	No consensus	**Consensus**	**Consensus**	
**Round 2**						
Mean score	7.8	7.3	7.1	**8.1**	**8.2**	7.7
Median score	9	7.5	7	**9**	**9**	
MADM	1.3	1.0	0.9	**0.9**	**0.8**	
Agreement	Moderate	High	High	**High**	**High**	
	No consensus	No consensus	No consensus	**Consensus**	**Consensus**	
**Round 3**						
Mean score	**8.3**	**7.8**	7.3	**8.2**	**8.3**	8.0
Median score	**9**	**8**	7.5	**9**	**9**	
MADM	**0.7**	**0.7**	1.0	**0.8**	**0.7**	
Agreement	**High**	**High**	High	**High**	**High**	
	**Consensus**	**Consensus**	No consensus	**Consensus**	**Consensus**	
**02- Child body composition**						
**Round 1**						
Mean score	6.5	6.4	6.7	6.5	7.2	6.7
Median score	7	7	7	7	7	
MADM	1.5	1.1	0.9	1.4	1.1	

Agreement	Low	Moderate	High	Moderate	High	
	No consensus	No consensus	No consensus	No consensus	No consensus	
**Round 2**						
Mean score	6.9	7.2	6.9	6.7	7.4	7.0
Median score	7	7	7	7	7	
MADM	1.2	0.5	0.9	1.0	1.1	
Agreement	Moderate	High	High	High	Moderate	
	No consensus	No consensus	No consensus	No consensus	No consensus	
**Round 3**						
Mean score	7.0	7.2	6.8	6.3	7.4	6.9
Median score	7	7	6.5	7	7	
MADM	1.2	0.6	1.2	1.1	1.0	
Agreement	Moderate	High	Moderate	Moderate	High	
	No consensus	No consensus	No consensus	No consensus	No consensus	
**03- Child circumference**						
**Round 1**						
Mean score	6.3	5.9	6.4	6.7	7.2	6.5
Median score	6	6	7	7	7	
MADM	1.4	1.1	1.2	1.2	1.2	
Agreement	Moderate	High	Moderate	Moderate	Moderate	
	No consensus	No consensus	No consensus	No consensus	No consensus	
**Round 2**						
Mean score	6.5	6.3	6.6	6.3	7.0	6.5
Median score	7	6	7	7	7	
MADM	1.1	0.7	0.4	0.9	1.1	
Agreement	Moderate	High	High	High	Moderate	
	No consensus	No consensus	No consensus	No consensus	No consensus	
**Round 3**						
Mean score	6.7	6.6	6.7	6.3	7.0	6.6

Median score	7	6	6.5	7	7	
MADM	0.7	0.6	0.7	1.1	1.0	
Agreement	High	High	High	Moderate	High	
	No consensus	No consensus	No consensus	No consensus	No consensus	
**04- Child screen time**						
**Round 1**						
Mean score	**7.7**	**7.7**	7.3	**7.9**	**7.5**	7.6
Median score	**8**	**8**	7	**9**	**8.0**	
MADM	**1.2**	**0.9**	1.4	**1.1**	**1.1**	
Agreement	**Moderate**	**High**	High	**High**	**Moderate**	
	**Consensus**	**Consensus**	No consensus	**Consensus**	**Consensus**	
**Round 2**						
Mean score	**8.0**	**7.7**	7.4	**8.1**	**7.7**	7.8
Median score	**8**	**8**	7	**9**	**8**	
MADM	**1.0**	**1.0**	1.3	**0.9**	**0.9**	
Agreement	**High**	**High**	Moderate	**High**	**High**	
	**Consensus**	**Consensus**	No consensus	**Consensus**	**Consensus**	
**Round 3**						
Mean score	**7.5**	**8.0**	**7.7**	**8.3**	**7.8**	7.9
Median score	**8**	**8**	**7.5**	**9**	**8**	
MADM	**0.8**	**0.7**	**1.0**	**0.7**	**0.9**	
Agreement	**High**	**High**	**High**	**High**	**High**	
	**Consensus**	**Consensus**	**Consensus**	**Consensus**	**Consensus**	
**05- Sedentary behaviour or physical activity home environment**						
**Round 1**						
Mean score	**7.6**	**7.4**	**7.8**	**7.8**	7.1	7.6
Median score	**8**	**8**	**8**	**9**	7	
MADM	**1.2**	**1.1**	**0.8**	**1.2**	1.2	
Agreement	**Moderate**	**High**	**High**	**Moderate**	Moderate	

	**Consensus**	**Consensus**	**Consensus**	**Consensus**	No consensus	
**Round 2**						
Mean score	**7.8**	**7.7**	**7.5**	**8.1**	7.4	7.7
Median score	**8**	**7**	**8**	**9**	8	
MADM	**1.0**	**0.9**	**0.7**	**0.9**	1.0	
Agreement	**High**	**High**	**High**	**High**	High	
	**Consensus**	**Consensus**	**Consensus**	**Consensus**	No consensus	
**Round 3**						
Mean score	**7.6**	**8.3**	**8.3**	**8.2**	**7.5**	8.0
Median score	**8**	**9**	**8**	**9**	**8**	
MADM	**1.1**	**0.7**	**0.3**	**0.8**	**1.0**	
Agreement	**High**	**High**	**High**	**High**	**High**	
	**Consensus**	**Consensus**	**Consensus**	**Consensus**	**Consensus**	
**06- Child time spent sedentary**						
**Round 1**						
Mean score	**7.7**	**7.8**	**8.1**	**7.9**	**7.4**	7.8
Median score	**8**	**8**	**8**	**9**	**8**	
MADM	**1.0**	**0.8**	**0.9**	**1.1**	**1.1**	
Agreement	**High**	**High**	**High**	**High**	**Moderate**	
	**Consensus**	**Consensus**	**Consensus**	**Consensus**	**Consensus**	
**Round 2**						
Mean score	**8.0**	**8.1**	**8.0**	**8.1**	**7.7**	8.0
Median score	**8**	**9**	**9**	**9**	**8**	
MADM	**0.8**	**0.9**	**1.0**	**0.9**	**0.9**	
Agreement	**High**	**High**	**High**	**High**	**High**	
	**Consensus**	**Consensus**	**Consensus**	**Consensus**	**Consensus**	
**Round 3**						
Mean score	**8.1**	**8.5**	**8.5**	**8.2**	**7.8**	8.2
Median score	**8**	**9**	**9**	**9**	**8**	

MADM	**0.9**	**0.5**	**0.5**	**0.8**	**0.8**	
Agreement	**High**	**High**	**High**	**High**	**High**	
	**Consensus**	**Consensus**	**Consensus**	**Consensus**	**Consensus**	
**07- Child active transport**						
**Round 1**						
Mean score	6.7	6.6	6.2	6.7	6.3	6.5
Median score	7	7	6	7	6	
MADM	1.1	1.2	1.2	1.2	1.3	
Agreement	Moderate	Moderate	Moderate	Moderate	Moderate	
	No consensus	No consensus	No consensus	No consensus	No consensus	
**Round 2**						
Mean score	6.7	6.8	6.7	6.9	6.4	6.7
Median score	7	7	7	7	7	
MADM	0.8	0.9	0.8	0.6	1.3	
Agreement	High	High	High	High	Moderate	
	No consensus	No consensus	No consensus	No consensus	No consensus	
**Round 3**						
Mean score	6.8	**7.3**	7.0	7.0	6.6	6.9
Median score	7	**8**	7	7	7	
MADM	0.7	**0.7**	1.3	0.7	1.1	
Agreement	High	**High**	Moderate	High	Moderate	
	No consensus	**Consensus**	No consensus	No consensus	No consensus	
**08- Child physical fitness**						
**Round 1**						
Mean score	6.6	6.5	6.6	6.7	6.4	6.6
Median score	7	6	6	7	7	
MADM	1.5	1.3	1.2	1.4	1.2	
Agreement	Low	Moderate	Moderate	Moderate	Moderate	
	No consensus	No consensus	No consensus	No consensus	No consensus	

**Round 2**						
Mean score	6.6	7.1	7.2	6.9	6.5	6.9
Median score	7	7	7	7	7	
MADM	1.2	0.8	0.7	0.8	1.1	
Agreement	Moderate	High	High	High	Moderate	
	No consensus	No consensus	No consensus	No consensus	No consensus	
**Round 3**						
Mean score	7.0	**7.3**	**7.2**	7.4	6.5	7.1
Median score	7	**8**	**7**	8	7	
MADM	1.1	**0.7**	**0.5**	0.8	1.1	
Agreement	High	**High**	**High**	High	Moderate	
	No consensus	**Consensus**	**Consensus**	No consensus	No consensus	
**09- Child active indoor play**						
**Round 1**						
Mean score	6.8	6.5	6.8	6.8	6.6	6.7
Median score	7	6	7	7	7	
MADM	1.2	1.3	1.1	1.3	1.1	
Agreement	Moderate	Moderate	High	Moderate	High	
	No consensus	No consensus	No consensus	No consensus	Non consensus	
**Round 2**						
Mean score	6.7	6.7	7.0	7.0	6.6	6.8
Median score	7	7	7	7	7	
MADM	1.0	1.4	0.9	1.0	0.9	
Agreement	High	Moderate	High	High	High	
	No consensus	No consensus	No consensus	No consensus	No consensus	
**Round 3**						
Mean score	6.5	**7.5**	**7.7**	7.2	6.7	7.1
Median score	6	**8**	**8**	7	7	
MADM	1.1	**0.5**	**1.0**	1.3	0.9	

Agreement	Moderate	**High**	**High**	Moderate	High	
	No consensus	**Consensus**	**Consensus**	No consensus	No consensus	
**10- Child active outdoor play**						
**Round 1**						
Mean score	7.3	7.3	7.1	7.2	7.0	7.2
Median score	7	8	7	7	7	
MADM	1.2	1.3	0.9	1.2	1.0	
Agreement	Moderate	Moderate	High	Moderate	High	
	No consensus	No consensus	No consensus	No consensus	No consensus	
**Round 2**						
Mean score	**7.3**	**7.7**	**7.6**	7.2	7.2	7.4
Median score	**7**	**8**	**8**	7.5	7	
MADM	**0.8**	**1.0**	**0.9**	1.1	0.9	
Agreement	**High**	**High**	**High**	High	High	
	**Consensus**	**Consensus**	**Consensus**	No consensus	No consensus	
**Round 3**						
Mean score	7.5	**8.0**	**8.5**	6.8	**7.3**	7.6
Median score	7	**8**	**9**	7	**7**	
MADM	1.3	**0.3**	**0.5**	1.6	**0.9**	
Agreement	Moderate	**High**	**High**	Low	**High**	
	No consensus	**Consensus**	**Consensus**	No consensus	**Consensus**	
**11- Child physical activity**						
**Round 1**						
Mean score	**7.7**	**7.7**	**7.9**	**8.1**	**7.7**	7.8
Median score	**8**	**8**	**9**	**8**	**8**	
MADM	**1.0**	**0.9**	**1.1**	**0.8**	**0.9**	
Agreement	**High**	**High**	**Moderate**	**High**	**High**	
	**Consensus**	**Consensus**	**Consensus**	**Consensus**	**Consensus**	
**Round 2**						

Mean score	**7.9**	**7.6**	**8.4**	**8.4**	**7.9**	8.0
Median score	**8**	**7**	**9**	**9**	**8**	2
MADM	**0.9**	**1.0**	**0.6**	**0.6**	**0.7**	
Agreement	**High**	**High**	**High**	**High**	**High**	
	**Consensus**	**Consensus**	**Consensus**	**Consensus**	**Consensus**	
**Round 3**						
Mean score	**8.0**	**8.0**	**8.8**	**8.4**	**8.1**	8.3
Median score	**9**	**8**	**9**	**9**	**8**	
MADM	**1.0**	**0.7**	**0.2**	**0.6**	**0.7**	
Agreement	**High**	**High**	**High**	**High**	**High**	
	**Consensus**	**Consensus**	**Consensus**	**Consensus**	**Consensus**	
**12- Infant tummy time**						
**Round 1**						
Mean score	6.60	7.47	6.39	6.61	5.93	6.6
Median score	7.0	8.0	6.5	6.0	6.0	
MADM	1.74	1.12	1.94	1.83	1.48	
Agreement	Low	Moderate	Low	Low	Low	
	No consensus	No consensus	No consensus	No consensus	No consensus	
**Round 2**						
Mean score	6.9	8.1	7.2	6.9	6.2	7.1
Median score	7	9	8	7	6	
MADM	1.4	1.3	1.4	1.6	1.4	
Agreement	Moderate	Moderate	Moderate	Low	Low	
	No consensus	No consensus	No consensus	No consensus	No consensus	
**Round 3**						
Mean score	6.7	7.8	**7.8**	7.3	6.3	7.2
Median score	7	8	**9**	9	7	
MADM	1.3	1.0	**1.2**	1.8	1.3	
Agreement	Moderate	High	**Moderate**	Low	Moderate	

	No consensus	No consensus	**Consensus**	No consensus	No consensus	
**13- Infant floor based play time**						
**Round 1**						
Mean score	6.7	7.4	6.8	6.7	6.0	6.7
Median score	7	8	7	6	6	
MADM	1.5	1.2	1.3	1.7	1.4	
Agreement	Low	Moderate	Moderate	Low	Moderate	
	No consensus	No consensus	No consensus	No consensus	No consensus	
**Round 2**						
Mean score	7.2	7.9	7.1	6.6	6.2	7.0
Median score	7	8	7	6	7	
MADM	1.1	0.9	1.2	1.5	1.3	
Agreement	Moderate	High	Moderate	Low	Moderate	
	No consensus	No consensus	No consensus	No consensus	No consensus	
**Round 3**						
Mean score	7.1	**8.0**	**8.7**	6.9	6.3	7.4
Median score	7	**8**	**9**	6	6	
MADM	1.1	**0.8**	**0.3**	1.8	1.1	
Agreement	Moderate	**High**	**High**	Low	Moderate	
	No consensus	**Consensus**	**Consensus**	No consensus	No consensus	
**14- Child enjoyment of physical activity and playing**						
**Round 1**						
Mean score	7.3	**8.0**	**7.8**	7.0	6.2	7.3
Median score	8	**9**	**8**	7	6	
MADM	1.4	**1.0**	**1.0**	1.6	1.2	
Agreement	Moderate	**High**	**High**	Low	Moderate	
	No consensus	**Consensus**	**Consensus**	No consensus	No consensus	
**Round 2**						
Mean score	**7.7**	**8.2**	7.7	6.9	6.2	7.3

Median score	**8**	**9**	8	7	6	
MADM	**1.1**	**0.8**	0.8	1.3	1.2	
Agreement	**Moderate**	**High**	High	Moderate	Moderate	
	**Consensus**	**Consensus**	No consensus	No consensus	No consensus	
**Round 3**						
Mean score	**7.7**	**8.5**	**8.2**	7.3	6.2	7.6
Median score	**8**	**9**	**9**	8	6	
MADM	**1.2**	**0.5**	**0.8**	1.3	1.3	
Agreement	**Moderate**	**High**	**High**	Moderate	Moderate	
	**Consensus**	**Consensus**	**Consensus**	No consensus	No consensus	
**15- Child sleep duration**						
**Round 1**						
Mean score	6.6	7.1	7.0	6.9	7.1	6.9
Median score	6	8	7	7	7	
MADM	1.1	1.1	1.4	1.7	1.0	
Agreement	High	Moderate	Moderate	Low	High	
	No consensus	No consensus	No consensus	No consensus	No consensus	
**Round 2**						
Mean score	6.7	7.3	**7.7**	7.3	7.2	7.2
Median score	7	7	**8**	8	7	
MADM	1.0	1.0	**0.8**	1.4	0.8	
Agreement	High	High	**High**	Low	High	
	No consensus	No consensus	**Consensus**	No consensus	Consensus	
**Round 3**						
Mean score	7.1	7.4	**7.7**	6.9	7.2	7.3
Median score	7	7	**8**	7	7	
MADM	0.9	0.8	**0.7**	1.4	0.8	
Agreement	High	High	**High**	Low	High	
	No consensus	No consensus	**Consensus**	No consensus	No consensus	

**16- Child sleep disturbance or problems**						
**Round 1**						
Mean score	6.8	7.2	6.4	6.1	6.4	6.6
Median score	7	8	7	7	6	
MADM	1.1	1.2	1.4	1.8	1.2	
Agreement	High	Moderate	Moderate	Low	Moderate	
	No consensus	No consensus	No consensus	No consensus	No consensus	
**Round 2**						
Mean score	6.8	7.2	**7.0**	6.2	6.6	6.7
Median score	7	7	**7**	7	6	
MADM	0.8	1.1	**0.7**	1.6	1.0	
Agreement	High	Moderate	**High**	Low	High	
	No consensus	No consensus	**Consensus**	No consensus	No consensus	
**Round 3**						
Mean score	7.1	7.2	6.8	5.8	6.5	6.7
Median score	7	7	7	7	6	
MADM	0.8	0.6	1.2	1.5	1.0	
Agreement	High	High	Moderate	Low	High	
	No consensus	No consensus	No consensus	No consensus	No consensus	
**17- Child sleep efficiency**						
**Round 1**						
Mean score	6.1	7.0	6.2	5.8	5.9	6.2
Median score	6	7	6	6	6	
MADM	1.2	1.1	1.2	1.7	1.1	
Agreement	Moderate	High	Moderate	Low	Moderate	
	No consensus	No consensus	No consensus	No consensus	No consensus	
**Round 2**						
Mean score	6.5	7.1	6.7	6.5	6.0	6.6
Median score	6	7	7	7	6	

MADM	0.9	0.6	1.1	1.5	1.0	
Agreement	High	High	Moderate	Low	High	
	No consensus	No consensus	No consensus	No consensus	No consensus	
**Round 3**						
Mean score	6.7	7.2	**7.3**	6.1	6.0	6.7
Median score	6	7	**8**	7	6	
MADM	0.8	1.0	**0.7**	1.6	1.0	
Agreement	High	High	**High**	Low	High	
	No consensus	No consensus	**Consensus**	No consensus	No consensus	
**18- Child sleep latency**						
**Round 1**						
Mean score	5.9	6.3	5.5	5.5	5.5	5.8
Median score	6	7	5	6	6	
MADM	1.2	1.2	1.4	1.6	1.2	
Agreement	Moderate	Moderate	Moderate	Low	Moderate	
	No consensus	No consensus	No consensus	No consensus	No consensus	
**Round 2**						
Mean score	6.0	6.4	5.4	5.7	5.4	5.8
Median score	6	7	5	6	5	
MADM	0.9	1.1	1.4	1.3	1.2	
Agreement	High	Moderate	Moderate	Moderate	Moderate	
	No consensus	No consensus	No consensus	No consensus	No consensus	
**Round 3**						
Mean score	6.3	6.8	6.6	5.5	5.3	6.1
Median score	6	7	7	6	5	
MADM	0.9	1.0	1.2	1.3	1.2	
Agreement	High	High	Moderate	Moderate	Moderate	
	No consensus	No consensus	No consensus	No consensus	No consensus	
**19- Child sleep patterns or timing**						

**Round 1**						
Mean score	6.3	6.1	6.2	5.9	6.6	6.2
Median score	6	7	6	6	7	
MADM	1.2	1.4	1.3	1.7	1.3	
Agreement	Moderate	Low	Moderate	Low	Moderate	
	No consensus	No consensus	No consensus	No consensus	No consensus	
**Round 2**						
Mean score	6.4	5.7	6.5	6.2	6.6	6.3
Median score	7	6	7	7	7	
MADM	1.2	1.0	1.0	1.2	1.3	
Agreement	Moderate	High	High	Moderate	Moderate	
	No consensus	No consensus	No consensus	No consensus	No consensus	
**Round 3**						
Mean score	7.1	5.8	6.8	6.2	6.6	6.5
Median score	7	6	7	6	7	
MADM	0.8	1.4	1.2	1.3	1.3	
Agreement	High	Moderate	Moderate	Moderate	Moderate	
	No consensus	No consensus	No consensus	No consensus	No consensus	
**20- Child sleep quality**						
**Round 1**						
Mean score	6.8	7.2	6.8	6.6	6.7	6.8
Median score	7	8	7	7	7	
MADM	1.2	1.1	1.3	1.4	1.2	
Agreement	Moderate	Moderate	Moderate	Low	Moderate	
	No consensus	No consensus	No consensus	No consensus	No consensus	
**Round 2**						
Mean score	7.1	7.1	**7.5**	7.0	6.7	7.1
Median score	7	7	**8**	7	7	
MADM	0.9	0.8	**1.0**	1.1	1.0	

Agreement	High	High	**High**	Moderate	High	
	No consensus	No consensus	**Consensus**	No consensus	No consensus	
**Round 3**						
Mean score	**7.4**	**7.2**	7.5	6.9	6.6	7.1
Median score	**7**	**7**	8	7	7	
MADM	**0.8**	**0.2**	0.8	1.7	0.9	
Agreement	**High**	**High**	High	Low	High	
	**Consensus**	**Consensus**	No consensus	No consensus	No consensus	
**21- Child carbohydrate intake or glycemic load**						
**Round 1**						
Mean score	6.4	6.6	6.4	5.9	5.6	6.2
Median score	6	7	6	6	6	
MADM	1.3	1.1	1.5	1.3	1.5	
Agreement	Moderate	High	Low	Moderate	Low	
	No consensus	No consensus	No consensus	No consensus	No consensus	
**Round 2**						
Mean score	6.3	7.3	6.1	5.8	5.6	6.2
Median score	6	8	6	6	6	
MADM	0.9	0.7	1.1	1.2	1.3	
Agreement	High	High	Moderate	Moderate	Moderate	
	No consensus	No consensus	No consensus	No consensus	No consensus	
**Round 3**						
Mean score	6.5	7.4	6.5	5.9	5.4	6.3
Median score	7	8	6	6	6	
MADM	1.0	0.6	1.2	1.2	1.2	
Agreement	High	High	Moderate	Moderate	Moderate	
	No consensus	No consensus	No consensus	No consensus	No consensus	
**22- Child dairy intake**						
**Round 1**						

Mean score	6.3	6.4	6.0	6.2	5.6	6.1
Median score	6	7	6	6	6	
MADM	1.2	1.2	1.5	1.3	1.3	
Agreement	Moderate	Moderate	Low	Moderate	Moderate	
	No consensus	No consensus	No consensus	No consensus	No consensus	
**Round 2**						
Mean score	6.5	6.9	5.6	6.4	5.6	6.2
Median score	7	7	6	6	6	
MADM	0.7	1.0	1.0	1.3	1.1	
Agreement	High	High	High	Moderate	Moderate	
	No consensus	No consensus	No consensus	No consensus	No consensus	
**Round 3**						
Mean score	6.5	7.4	6.2	6.8	5.4	6.5
Median score	7	8	6	6	6	
MADM	0.9	0.6	0.5	1.4	1.2	
Agreement	High	High	High	Low	Moderate	
	No consensus	No consensus	No consensus	No consensus	No consensus	
**23- Child diet quality**						
**Round 1**						
Mean score	**7.7**	**8.1**	**8.1**	**8.0**	**7.7**	7.9
Median score	**8**	**8**	**9**	**8**	**8**	
MADM	**1.0**	**0.6**	**0.9**	**0.9**	**0.9**	
Agreement	**High**	**High**	**High**	**High**	**High**	
	**Consensus**	**Consensus**	**Consensus**	**Consensus**	**Consensus**	
**Round 2**						
Mean score	**8.3**	**8.4**	**8.1**	**8.1**	**7.8**	8.2
Median score	**9**	**9**	**8**	**8**	**8**	
MADM	**0.7**	**0.6**	**0.7**	**0.7**	**0.8**	
Agreement	**High**	**High**	**High**	**High**	**High**	

	**Consensus**	**Consensus**	**Consensus**	**Consensus**	**Consensus**	
**Round 3**						
Mean score	**8.5**	**8.8**	**8.8**	**8.3**	**8.0**	8.5
Median score	**9**	**9**	**9**	**8**	**8**	
MADM	**0.5**	**0.2**	**0.2**	**0.5**	**0.7**	
Agreement	**High**	**High**	**High**	**High**	**High**	
	**Consensus**	**Consensus**	**Consensus**	**Consensus**	**Consensus**	
**24- Child dietary intake**						
**Round 1**						
Mean score	**7.3**	**7.7**	**8.1**	**8.1**	**7.5**	7.7
Median score	**7**	**8**	**9**	**9**	**8**	
MADM	**1.2**	**0.8**	**1.0**	**0.9**	**1.1**	
Agreement	**Moderate**	**High**	**High**	**High**	**High**	
	**Consensus**	**Consensus**	**Consensus**	**Consensus**	**Consensus**	
**Round 2**						
Mean score	**8.0**	**8.0**	**8.2**	**8.3**	**7.6**	8.0
Median score	**8**	**8**	**9**	**9**	**8**	
MADM	**0.9**	**0.8**	**0.8**	**0.7**	**1.0**	
Agreement	**High**	**High**	**High**	**High**	**High**	
	**Consensus**	**Consensus**	**Consensus**	**Consensus**	**Consensus**	
**Round 3**						
Mean score	**8.3**	**8.3**	**8.3**	**8.6**	**7.7**	8.2
Median score	**9**	**9**	**9**	**9**	**8**	
MADM	**0.7**	**0.7**	**0.7**	**0.4**	**0.9**	
Agreement	**High**	**High**	**High**	**High**	**High**	
	**Consensus**	**Consensus**	**Consensus**	**Consensus**	**Consensus**	
**25- Child fat intake**						
**Round 1**						
Mean score	6.5	7.1	6.9	6.8	6.1	6.7

Median score	7	7	7	6	6	
MADM	1.2	1.2	1.4	1.3	1.2	
Agreement	Moderate	Moderate	Moderate	Moderate	Moderate	
	No consensus	No consensus	No consensus	No consensus	No consensus	
**Round 2**						
Mean score	6.6	**7.6**	6.9	6.9	6.1	6.8
Median score	6	**7**	7	7	6	
MADM	1.0	**0.6**	0.5	0.9	1.1	
Agreement	High	**High**	High	High	Moderate	
	No consensus	**Consensus**	No consensus	No consensus	No consensus	
**Round 3**						
Mean score	6.5	**8.0**	6.8	7.2	5.9	6.9
Median score	6	**8**	7	7	6	
MADM	0.8	**0.8**	0.8	0.9	1.1	
Agreement	High	**High**	High	High	Moderate	
	No consensus	**Consensus**	No consensus	No consensus	No consensus	
**26- Child fibre intake**						
**Round 1**						
Mean score	6.6	6.9	6.7	6.6	6.3	6.6
Median score	7	7	7	6	6	
MADM	1.2	1.3	1.5	1.3	1.2	
Agreement	Moderate	Moderate	Low	Moderate	Moderate	
	No consensus	No consensus	No consensus	No consensus	No consensus	
**Round 2**						
Mean score	6.6	7.6	6.3	6.6	6.1	6.6
Median score	6	8	6	6	6	
MADM	0.9	0.9	0.9	1.1	1.1	
Agreement	High	High	High	High	Moderate	
	No consensus	No consensus	No consensus	No consensus	No consensus	

**Round 3**						
Mean score	6.8	7.8	6.8	6.4	6.0	6.8
Median score	7	8	7	6	6	
MADM	0.9	0.6	0.8	1.1	1.1	
Agreement	High	High	High	Moderate	Moderate	
	No consensus	Consensus	No consensus	No consensus	No consensus	
**27- Child fruit and vegetable intake**						
**Round 1**						
Mean score	7.3	**7.3**	**7.9**	7.7	**7.2**	7.5
Median score	7	**8**	**8**	9	**7**	
MADM	1.2	**1.1**	**1.0**	1.3	**1.0**	
Agreement	Moderate	**Moderate**	**High**	Moderate	**High**	
	No consensus	**Consensus**	**Consensus**	No consensus	**Consensus**	
**Round 2**						
Mean score	**7.8**	**8.4**	**8.0**	8.0	**7.3**	7.9
Median score	**8**	**9**	**8**	9	**7**	
MADM	**0.8**	**0.6**	**0.8**	1.0	**0.8**	
Agreement	**High**	**High**	**High**	High	**High**	
	**Consensus**	**Consensus**	**Consensus**	No consensus	**Consensus**	
**Round 3**						
Mean score	**7.9**	**9.0**	**8.2**	8.0	**7.3**	8.1
Median score	**8**	**9**	**8**	9	**7**	
MADM	**0.8**	**0.0**	**0.5**	1.0	**0.8**	
Agreement	**High**	**High**	**High**	High	**High**	
	**Consensus**	**Consensus**	**Consensus**	No consensus	**Consensus**	
**28- Child grains; breads; cereals intake**						
**Round 1**						
Mean score	6.4	6.9	7.1	6.9	6.1	6.7
Median score	6	7	7	7	6	

MADM	1.0	1.2	1.2	1.1	1.3	
Agreement	High	Moderate	Moderate	Moderate	Moderate	
	No consensus	No consensus	No consensus	No consensus	No consensus	
**Round 2**						
Mean score	6.5	7.3	6.8	6.9	6.1	6.7
Median score	6	8	7	7	6	
MADM	0.9	0.8	0.7	1.2	1.1	
Agreement	High	High	High	Moderate	Moderate	
	No consensus	No consensus	No consensus	No consensus	No consensus	
**Round 3**						
Mean score	6.6	**7.6**	6.8	7.0	5.9	6.8
Median score	6	**8**	7	8	6	
MADM	0.9	**0.4**	0.8	1.4	1.1	
Agreement	High	**High**	High	Low	High	
	No consensus	**Consensus**	No consensus	No consensus	No consensus	
**29- Child meat; poultry; fish intake**						
**Round 1**						
Mean score	6.3	6.3	7.1	6.7	5.9	6.5
Median score	6	6	7	6	6	
MADM	1.0	1.7	1.4	1.3	1.3	
Agreement	High	Low	Moderate	Moderate	Moderate	
	No consensus	No consensus	No consensus	No consensus	No consensus	
**Round 2**						
Mean score	6.4	7.3	6.8	6.8	5.8	6.6
Median score	6	8	7	7	6	
MADM	0.9	1.3	0.6	1.5	1.2	
Agreement	High	Moderate	High	Low	Moderate	
	No consensus	No consensus	No consensus	No consensus	No consensus	
**Round 3**						

Mean score	6.5	7.4	7.0	7.3	5.6	6.8
Median score	6	8	7	8	5	
MADM	0.6	1.4	1.0	1.6	1.1	
Agreement	High	Moderate	High	Low	Moderate	
	No consensus	No consensus	No consensus	No consensus	No consensus	
**30- Child non-core food intake**						
**Round 1**						
Mean score	7.3	6.9	7.4	**7.6**	6.8	7.2
Median score	8	8	8	**8**	7	
MADM	1.4	1.7	1.2	**1.2**	1.3	
Agreement	Low	Low	Moderate	**Moderate**	Moderate	
	No consensus	No consensus	No consensus	**Consensus**	No consensus	
**Round 2**						
Mean score	**8.0**	**7.8**	7.3	**7.7**	7.1	7.6
Median score	**9**	**9**	8	**9**	7	
MADM	**1.0**	**1.2**	1.3	**1.3**	1.1	
Agreement	**High**	**Moderate**	Moderate	**Moderate**	High	
	**Consensus**	**Consensus**	No consensus	**Consensus**	No consensus	
**Round 3**						
Mean score	**8.0**	7.7	**7.5**	**8.1**	7.1	7.7
Median score	**9**	9	**8**	**9**	7	
MADM	**1.0**	1.3	**0.8**	**0.9**	1.0	
Agreement	**High**	Moderate	**High**	**High**	High	
	**Consensus**	No consensus	**Consensus**	**Consensus**	No consensus	
**31- Child non-core beverages intake**						
**Round 1**						
Mean score	**7.9**	**7.1**	7.6	**7.7**	**7.3**	7.5
Median score	**9**	**8**	8	**8**	**7**	
MADM	**1.1**	**1.5**	1.2	**1.2**	**1.0**	

Agreement	**Moderate**	**Low**	Moderate	**Moderate**	**High**	
	**Consensus**	**Consensus**	No consensus	**Consensus**	**Consensus**	
**Round 2**						
Mean score	**8.5**	**8.0**	7.3	**8.3**	**7.5**	7.9
Median score	**9**	**9**	8	**9**	**8**	
MADM	**0.5**	**1.0**	1.3	**0.7**	**0.9**	
Agreement	**High**	**High**	Moderate	**High**	**High**	
	**Consensus**	**Consensus**	No consensus	**Consensus**	**Consensus**	
**Round 3**						
Mean score	**8.5**	**8.0**	**7.5**	**8.2**	**7.6**	8.0
Median score	**9**	**9**	**8**	**9**	**7**	
MADM	**0.5**	**1.0**	**0.8**	**0.8**	**0.9**	
Agreement	**High**	**High**	**High**	**High**	**High**	
	**Consensus**	**Consensus**	**Consensus**	**Consensus**	**Consensus**	
**32- Child out of home foods intake**						
**Round 1**						
Mean score	7.2	6.5	6.8	**7.4**	6.5	6.9
Median score	7	7	7	**7**	7	
MADM	1.3	1.4	1.0	**0.9**	1.3	
Agreement	Moderate	Low	High	**High**	Moderate	
	No consensus	No consensus	No consensus	**Consensus**	No consensus	
**Round 2**						
Mean score	7.6	**7.6**	6.8	**7.6**	6.8	7.3
Median score	8	**7**	7	**7**	7	
MADM	1.2	**0.8**	0.4	**0.6**	0.9	
Agreement	Moderate	**High**	High	**High**	High	
	No consensus	**Consensus**	No consensus	**Consensus**	No consensus	
**Round 3**						
Mean score	**7.5**	**7.7**	6.7	**8.0**	6.7	7.3

Median score	**8**	**8**	7	**8**	7	
MADM	**0.9**	**1.0**	0.7	**0.7**	0.9	
Agreement	**High**	**High**	High	**High**	High	
	**Consensus**	**Consensus**	No consensus	**Consensus**	No consensus	
**33- Child protein intake**						
**Round 1**						
Mean score	6.5	6.6	6.8	6.6	5.7	6.4
Median score	6	7	7	6	6	
MADM	1.0	1.3	1.2	1.1	1.4	
Agreement	High	Moderate	Moderate	Moderate	Low	
	No consensus	No consensus	No consensus	No consensus	No consensus	
**Round 2**						
Mean score	6.6	7.3	6.8	6.5	5.7	6.6
Median score	6	8	7	6	6	
MADM	0.8	1.3	1.0	1.4	1.3	
Agreement	High	Moderate	High	Moderate	Moderate	
	No consensus	No consensus	No consensus	No consensus	No consensus	
**Round 3**						
Mean score	6.9	7.8	7.2	6.2	5.6	6.7
Median score	7	8	8	6	5	
MADM	0.9	1.0	0.8	1.3	1.2	
Agreement	High	High	High	Moderate	Moderate	
	No consensus	No consensus	No consensus	No consensus	No consensus	
**34- Child sodium intake**						
**Round 1**						
Mean score	6.4	6.7	6.4	6.5	5.6	6.3
Median score	7	7	7	6	6	
MADM	1.4	1.1	1.6	1.3	1.4	
Agreement	Low	Moderate	Low	Moderate	Low	

	No consensus	No consensus	No consensus	No consensus	No consensus	
**Round 2**						
Mean score	6.5	**7.2**	5.6	6.3	5.5	6.2
Median score	7	**7**	6	6	6	
MADM	1.2	**0.4**	1.2	1.4	1.3	
Agreement	Moderate	**High**	Moderate	Low	Moderate	
	No consensus	**Consensus**	No consensus	No consensus	No consensus	
**Round 3**						
Mean score	6.7	**7.6**	6.3	6.9	5.4	6.6
Median score	7	**8**	6	7	6	
MADM	1.1	**0.4**	0.7	1.4	1.3	
Agreement	High	**High**	High	Low	Moderate	
	No consensus	**Consensus**	No consensus	No consensus	No consensus	
**35- Child total and added sugars intake**						
**Round 1**						
Mean score	**7.6**	7.0	**7.8**	**7.8**	7.2	7.5
Median score	**8**	7	**8**	**8**	7	
MADM	**1.1**	1.2	**1.2**	**0.9**	1.2	
Agreement	**Moderate**	Moderate	**Moderate**	**High**	Moderate	
	**Consensus**	No consensus	**Consensus**	**Consensus**	No consensus	
**Round 2**						
Mean score	**8.2**	7.8	7.3	**8.0**	7.3	7.7
Median score	**8**	8	8	**8**	8	
MADM	**0.7**	0.9	1.1	**0.9**	1.0	
Agreement	**High**	High	Moderate	**High**	High	
	**Consensus**	Consensus	No consensus	**Consensus**	No consensus	
**Round 3**						
Mean score	**8.2**	8.0	**7.5**	**8.0**	7.3	7.8
Median score	**9**	8	**8**	**8**	8	

MADM	**0.8**	0.8	**0.5**	**0.9**	1.1	
Agreement	**High**	High	**High**	**High**	Moderate	
	**Consensus**	No consensus	**Consensus**	**Consensus**	No consensus	
**36- Child desire for drinks**						
**Round 1**						
Mean score	6.7	6.8	6.5	5.7	5.6	6.3
Median score	7	7	7	6	6	
MADM	1.6	1.3	1.6	1.5	1.6	
Agreement	Low	Moderate	Low	Low	Low	
	No consensus	No consensus	No consensus	No consensus	No consensus	
**Round 2**						
Mean score	6.8	7.6	5.8	6.1	5.5	6.4
Median score	7	8	6	6	6	
MADM	1.2	0.9	2.0	1.2	1.6	
Agreement	Moderate	High	Low	Moderate	Low	
	No consensus	No consensus	No consensus	No consensus	No consensus	
**Round 3**						
Mean score	6.9	**7.8**	6.2	6.3	5.4	6.5
Median score	7	**8**	6	6	6	
MADM	1.2	**0.6**	1.5	1.4	1.6	
Agreement	Moderate	**High**	Low	Low	Low	
	No consensus	**Consensus**	No consensus	No consensus	No consensus	
**37- Child eating in response to emotion**						
**Round 1**						
Mean score	7.3	7.1	7.1	6.4	6.2	6.8
Median score	7	7	7	6	6	
MADM	1.4	1.2	1.5	1.6	1.3	
Agreement	Moderate	Moderate	Low	Low	Moderate	
	No consensus	No consensus	No consensus	No consensus	No consensus	

**Round 2**						
Mean score	7.6	7.7	7.0	6.8	6.2	7.0
Median score	8	8	7	7	6	
MADM	1.1	0.8	1.0	1.5	1.0	
Agreement	Moderate	High	High	Low	High	
	No consensus	No consensus	No consensus	No consensus	No consensus	
**Round 3**						
Mean score	**8.0**	**7.7**	7.2	7.3	6.2	7.3
Median score	**9**	**8**	7	8	6	
MADM	**1.0**	**0.7**	0.8	1.3	1.0	
Agreement	**High**	**High**	High	Moderate	High	
	**Consensus**	**Consensus**	No consensus	No consensus	No consensus	
**38- Child enjoyment of food**						
**Round 1**						
Mean score	6.7	6.8	**7.2**	6.5	6.0	6.6
Median score	7	7	**7**	7	6	
MADM	1.4	1.3	**0.9**	1.9	1.4	
Agreement	Low	Moderate	**High**	Low	Low	
	No consensus	No consensus	**Consensus**	No consensus	No consensus	
**Round 2**						
Mean score	6.9	**7.6**	6.7	6.9	6.0	6.8
Median score	7	**8**	7	7	6	
MADM	1.4	**1.0**	0.9	1.4	1.2	
Agreement	Moderate	**High**	High	Low	Moderate	
	No consensus	**Consensus**	No consensus	No consensus	No consensus	
**Round 3**						
Mean score	6.9	**8.3**	7.3	7.0	6.0	7.1
Median score	6	**8**	8	8	6	
MADM	1.3	**0.3**	1.0	1.7	1.1	

Agreement	Moderate	**High**	High	Low	Moderate	
	No consensus	**Consensus**	No consensus	No consensus	No consensus	
**39- Child meal patterns**						
**Round 1**						
Mean score	7.3	7.0	**7.2**	7.1	6.8	7.1
Median score	7	7	**8**	7	7	
MADM	1.1	1.1	**1.3**	1.4	1.2	
Agreement	Moderate	Moderate	**Moderate**	Moderate	Moderate	
	No consensus	No consensus	**Consensus**	No consensus	No consensus	
**Round 2**						
Mean score	**7.6**	**7.6**	6.7	**7.3**	6.9	7.2
Median score	**7**	**8**	7	**7**	7	
MADM	**0.9**	**0.9**	1.1	**1.0**	0.9	
Agreement	**High**	**High**	Moderate	**High**	High	
	**Consensus**	**Consensus**	No consensus	**Consensus**	No consensus	
**Round 3**						
Mean score	**7.3**	**8.0**	**7.3**	**7.6**	6.9	7.4
Median score	**7**	**8**	**8**	**8**	7	
MADM	**0.9**	**0.4**	**0.7**	**0.9**	0.9	
Agreement	**High**	**High**	**High**	**High**	High	
	**Consensus**	**Consensus**	**Consensus**	**Consensus**	No consensus	
**40- Child food neophobia**						
**Round 1**						
Mean score	6.8	6.4	6.6	5.4	5.8	6.2
Median score	7	7	7	5	6	
MADM	1.7	1.5	1.2	1.4	1.3	
Agreement	Low	Low	Moderate	Moderate	Moderate	
	No consensus	No consensus	No consensus	No consensus	No consensus	
**Round 2**						

Mean score	7.3	7.2	**7.2**	5.7	5.8	6.6
Median score	7	7	**7**	5	6	
MADM	1.2	0.9	**1.0**	1.0	1.2	
Agreement	Moderate	High	**High**	High	Moderate	
	No consensus	No consensus	**Consensus**	No consensus	No consensus	
**Round 3**						
Mean score	7.4	**7.5**	7.4	6.3	5.6	6.8
Median score	8	**8**	7	6	6	
MADM	1.3	**0.5**	1.2	1.1	1.2	
Agreement	Moderate	**High**	Moderate	Moderate	Moderate	
	No consensus	**Consensus**	No consensus	No consensus	No consensus	
**41- Child satiety responsiveness**						
**Round 1**						
Mean score	6.7	7.2	7.2	6.3	6.3	6.8
Median score	7	8	7	6	6	
MADM	1.4	1.3	1.2	1.5	1.3	
Agreement	Moderate	Moderate	Moderate	Low	Moderate	
	No consensus	No consensus	No consensus	No consensus	No consensus	
**Round 2**						
Mean score	**7.5**	7.9	7.1	6.8	6.6	7.2
Median score	**7**	9	7	7	7	
MADM	**1.1**	1.1	1.1	0.9	1.1	
Agreement	**Moderate**	Moderate	Moderate	High	Moderate	
	**Consensus**	No consensus	No consensus	No consensus	No consensus	
**Round 3**						
Mean score	**7.8**	**8.4**	**7.5**	7.0	6.5	7.4
Median score	**9**	**9**	**7**	7	6	
MADM	**1.2**	**0.6**	**0.8**	0.9	1.1	
Agreement	**Moderate**	**High**	**High**	High	High	

	**Consensus**	**Consensus**	**Consensus**	No consensus	No consensus	
**42- Child slowness in eating**						
**Round 1**						
Mean score	5.6	5.4	6.1	5.5	5.2	5.6
Median score	6	5	6	6	5	
MADM	1.6	1.6	1.3	1.4	1.5	
Agreement	Low	Low	Moderate	Moderate	Low	
	No consensus	No consensus	No consensus	No consensus	No consensus	
**Round 2**						
Mean score	5.5	6.7	5.5	5.6	5.1	5.7
Median score	6	7	5	5	5	
MADM	0.9	1.2	0.8	1.1	1.5	
Agreement	High	Moderate	High	High	Low	
	No consensus	No consensus	No consensus	No consensus	No consensus	
**Round 3**						
Mean score	5.5	7.2	5.7	5.6	4.8	5.7
Median score	6	7	5	5	5	
MADM	1.2	1.2	0.7	1.4	1.4	
Agreement	Moderate	Moderate	High	Low	Moderate	
	No consensus	No consensus	No consensus	No consensus	No consensus	
**43- Parent/caregiver weight based anthropometry**						
**Round 1**						
Mean score	6.5	6.4	6.6	6.6	7.0	6.6
Median score	7	6	7	7	7	
MADM	1.2	1.5	1.2	1.1	1.2	
Agreement	Moderate	Low	Moderate	Moderate	Moderate	
	No consensus	No consensus	No consensus	No consensus	No consensus	
**Round 2**						
Mean score	6.9	6.4	6.5	6.9	7.0	6.7

Median score	7	7	7	7	7	
MADM	0.7	0.9	1.0	1.0	0.9	
Agreement	High	High	High	High	High	
	No consensus	No consensus	No consensus	No consensus	No consensus	
**Round 3**						
Mean score	7.1	6.8	6.8	6.8	7.1	6.9
Median score	7	7	7	7	7	
MADM	0.8	0.5	0.6	0.7	0.8	
Agreement	High	High	High	High	High	
	No consensus	Consensus	No consensus	No consensus	No consensus	
**44- Parent/caregiver screen time parenting practice**						
**Round 1**						
Mean score	**7.3**	6.6	**6.9**	6.5	6.4	6.7
Median score	**7**	7	**7**	7	7	
MADM	**1.0**	1.5	**0.8**	1.5	1.1	
Agreement	**High**	Low	**High**	Low	Moderate	
	**Consensus**	No consensus	**Consensus**	No consensus	No consensus	
**Round 2**						
Mean score	**7.5**	7.6	**7.0**	6.4	6.7	7.0
Median score	**8**	8	**7**	7	7	
MADM	**0.8**	1.1	**0.7**	1.4	1.1	
Agreement	**High**	Moderate	**High**	Moderate	Moderate	
	**Consensus**	No consensus	**Consensus**	No consensus	No consensus	
**Round 3**						
Mean score	7.2	**8.2**	**7.6**	6.9	6.9	7.4
Median score	8	**8**	**8**	7	7	
MADM	1.1	**0.5**	**0.4**	1.2	1.0	
Agreement	High	**High**	**High**	Moderate	High	
	No consensus	**Consensus**	**Consensus**	No consensus	No consensus	

**45- Parent/caregiver role modelling of sedentary behaviours**						
**Round 1**						
Mean score	**7.6**	**7.2**	**7.5**	6.9	6.6	7.2
Median score	**8**	**8**	**8**	7	7	
MADM	**0.9**	**1.0**	**0.9**	1.4	1.2	
Agreement	**High**	**High**	**High**	Moderate	Moderate	
	**Consensus**	**Consensus**	**Consensus**	No consensus	No consensus	
**Round 2**						
Mean score	**7.8**	**7.3**	**7.6**	6.5	6.8	7.2
Median score	**8**	**8**	**8**	7	7	
MADM	**0.7**	**1.1**	**0.4**	1.2	1.1	
Agreement	**High**	**Moderate**	**High**	Moderate	High	
	**Consensus**	**Consensus**	**Consensus**	No consensus	No consensus	
**Round 3**						
Mean score	**7.8**	**8.3**	**7.4**	6.6	7.0	7.4
Median score	**8**	**8**	**7**	7	7	
MADM	**1.0**	**0.2**	**0.4**	1.1	0.9	
Agreement	**High**	**High**	**High**	Moderate	High	
	**Consensus**	**Consensus**	**Consensus**	No consensus	No consensus	
**46- Parent/caregiver role modelling of physical activity**						
**Round 1**						
Mean score	**7.7**	**7.4**	**7.6**	6.9	6.9	7.3
Median score	**8**	**8**	**8**	7	7	
MADM	**0.9**	**1.1**	**0.7**	1.3	1.1	
Agreement	**High**	**High**	**High**	Moderate	Moderate	
	**Consensus**	**Consensus**	**Consensus**	No consensus	No consensus	
**Round 2**						
Mean score	**7.8**	**7.3**	**7.7**	7.1	**7.0**	7.4
Median score	**8**	**8**	**8**	8	**7**	

MADM	**0.8**	**1.1**	**0.3**	1.1	**0.9**	
Agreement	**High**	**Moderate**	**High**	High	**High**	
	**Consensus**	**Consensus**	**Consensus**	No consensus	**Consensus**	
**Round 3**						
Mean score	**7.8**	**8.1**	**8.2**	7.3	**7.2**	7.7
Median score	**8**	**8**	**8**	8	**7**	
MADM	**1.1**	**0.3**	**0.2**	1.1	**0.7**	
Agreement	**Moderate**	**High**	**High**	Moderate	**High**	
	**Consensus**	**Consensus**	**Consensus**	No consensus	**Consensus**	
**47- Parent/caregiver role modelling of healthy eating**						
**Round 1**						
Mean score	**8.1**	**7.7**	**7.9**	7.1	7.0	7.6
Median score	**9**	**8**	**8**	7	7	
MADM	**0.9**	**0.7**	**0.6**	1.2	1.0	
Agreement	**High**	**High**	**High**	Moderate	High	
	**Consensus**	**Consensus**	**Consensus**	No consensus	No consensus	
**Round 2**						
Mean score	**8.4**	**7.7**	**8.2**	**7.3**	**7.3**	7.8
Median score	**9**	**8**	**8**	**8**	**7**	
MADM	**0.6**	**0.7**	**0.5**	**1.0**	**0.9**	
Agreement	**High**	**High**	**High**	**High**	**High**	
	**Consensus**	**Consensus**	**Consensus**	**Consensus**	**Consensus**	
**Round 3**						
Mean score	**8.4**	**8.3**	**8.6**	**7.8**	**7.5**	8.1
Median score	**9**	**8**	**9**	**8**	**7**	
MADM	**0.6**	**0.2**	**0.4**	**0.9**	**0.9**	
Agreement	**High**	**High**	**High**	**High**	**High**	
	**Consensus**	**Consensus**	**Consensus**	**Consensus**	**Consensus**	
**48- Parent/caregiver encouragement of physical activity**						

**Round 1**						
Mean score	**7.6**	**7.6**	**7.5**	6.8	6.6	7.2
Median score	**7**	**8**	**8**	7	7	
MADM	**1.0**	**0.7**	**0.9**	1.7	1.1	
Agreement	**High**	**High**	**High**	Low	Moderate	
	**Consensus**	**Consensus**	**Consensus**	No consensus	No consensus	
**Round 2**						
Mean score	**7.6**	**7.6**	**7.5**	7.4	6.8	7.4
Median score	**8**	**8**	**8**	8	7	
MADM	**0.9**	**0.8**	**0.6**	1.4	1.0	
Agreement	**High**	**High**	**High**	Moderate	High	
	**Consensus**	**Consensus**	**Consensus**	No consensus	No consensus	
**Round 3**						
Mean score	**7.8**	**8.3**	**7.8**	7.7	**6.9**	7.7
Median score	**8**	**8**	**8**	8	**7**	
MADM	**0.8**	**0.2**	**1.0**	1.0	**0.7**	
Agreement	**High**	**High**	**High**	High	**High**	
	**Consensus**	**Consensus**	**Consensus**	No consensus	**Consensus**	
**49- Parent/caregiver co-participation in physical activity**						
**Round 1**						
Mean score	**7.3**	**7.5**	**7.5**	6.7	6.6	7.1
Median score	**7**	**8**	**8**	7	7	
MADM	**1.1**	**1.1**	**1.1**	1.4	1.1	
Agreement	**Moderate**	**Moderate**	**Moderate**	Low	Moderate	
	**Consensus**	**Consensus**	**Consensus**	No consensus	No consensus	
**Round 2**						
Mean score	**7.5**	**7.7**	7.5	6.9	6.8	7.3
Median score	**8**	**8**	8	7	7	
MADM	**1.1**	**0.9**	1.1	0.9	1.0	

Agreement	**Moderate**	**High**	Moderate	High	High	
	**Consensus**	**Consensus**	No consensus	No consensus	No consensus	
**Round 3**						
Mean score	**7.5**	**8.3**	**8.0**	7.3	6.9	7.6
Median score	**8**	**8**	**8**	8	7	
MADM	**1.1**	**0.2**	**0.8**	0.9	1.0	
Agreement	**High**	**High**	**High**	High	High	
	**Consensus**	**Consensus**	**Consensus**	No consensus	No consensus	
**50- Parent/caregiver physical activity parenting practice**						
**Round 1**						
Mean score	7.11627907	7.105263158	**7.235294118**	6.578947368	6.481012658	6.9
Median score	7	8	**8**	7	7	
MADM	1.23255814	1.105263158	**1.117647059**	1.368421053	1.17721519	
Agreement	Moderate	Moderate	**Moderate**	Moderate	Moderate	
	No consensus	No consensus	**Consensus**	No consensus	No consensus	
**Round 2**						
Mean score	7.4	**7.4**	**7.5**	6.9	6.6	7.2
Median score	7	**8**	**8**	7	7	
MADM	1.0	**1.0**	**0.5**	1.2	1.2	
Agreement	High	**High**	**High**	Moderate	Moderate	
	No consensus	**Consensus**	**Consensus**	No consensus	No consensus	
**Round 3**						
Mean score	**7.3**	**8.0**	**7.6**	7.3	6.7	7.4
Median score	**7**	**8**	**8**	8	7	
MADM	**1.1**	**0.5**	**0.4**	1.1	1.0	
Agreement	**High**	**High**	**High**	Moderate	High	
	**Consensus**	**Consensus**	**Consensus**	No consensus	No consensus	
**51- Parent/caregiver sleep parenting practice**						
**Round 1**						

Mean score	6.6	6.9	6.4	5.8	6.1	6.4
Median score	7	7	7	6	6	
MADM	1.1	1.2	1.3	2.2	1.2	
Agreement	Moderate	Moderate	Moderate	Low	Moderate	
	No consensus	No consensus	No consensus	No consensus	No consensus	
**Round 2**						
Mean score	6.5	7.5	6.4	6.2	6.1	6.5
Median score	6	8	7	7	6	
MADM	1.1	0.8	0.8	1.7	1.0	
Agreement	Moderate	High	High	Low	High	
	No consensus	No consensus	No consensus	No consensus	No consensus	
**Round 3**						
Mean score	6.7	7.8	7.2	6.8	6.3	7.0
Median score	7	8	7	7	6	
MADM	0.9	0.5	1.0	1.5	0.9	
Agreement	High	High	High	Low	High	
	No consensus	No consensus	No consensus	No consensus	No consensus	
**52- Parent/caregiver daytime dysfunction or fatigue**						
**Round 1**						
Mean score	6.7	7.0	5.7	5.2	5.3	6.0
Median score	7	7	5	4	6	
MADM	1.2	1.2	2.0	2.2	1.4	
Agreement	Moderate	Moderate	Low	Low	Moderate	
	No consensus	No consensus	No consensus	No consensus	No consensus	
**Round 2**						
Mean score	6.7	7.3	6.1	5.3	5.3	6.1
Median score	7	8	6	6	6	
MADM	1.2	1.0	1.5	1.3	1.4	
Agreement	Moderate	High	Low	Moderate	Moderate	

	No consensus	No consensus	No consensus	No consensus	No consensus	
**Round 3**						
Mean score	6.7	7.7	7.0	6.0	5.2	6.5
Median score	7	8	7	6	6	
MADM	1.1	0.7	1.2	0.8	1.4	
Agreement	Moderate	High	Moderate	High	Moderate	
	No consensus	No consensus	No consensus	No consensus	No consensus	
**53- Parent/caregiver nutrition parenting practices**						
**Round 1**						
Mean score	**7.7**	7.2	**7.8**	6.9	6.7	7.3
Median score	**8**	8	**8**	7	7	
MADM	**1.0**	1.0	**0.9**	1.5	1.1	
Agreement	**High**	High	**High**	Low	Moderate	
	**Consensus**	No consensus	**Consensus**	No consensus	No consensus	
**Round 2**						
Mean score	**7.8**	**7.6**	**7.4**	7.1	6.9	7.4
Median score	**8**	**8**	**7**	7	7	
MADM	**0.7**	**0.8**	**0.7**	1.2	0.8	
Agreement	**High**	**High**	**High**	Moderate	High	
	**Consensus**	**Consensus**	**Consensus**	No consensus	No consensus	
**Round 3**						
Mean score	**8.2**	**7.9**	**8.0**	7.3	7.1	7.7
Median score	**8**	**8**	**9**	8	7	
MADM	**0.7**	**0.7**	**1.0**	1.1	0.8	
Agreement	**High**	**High**	**High**	Moderate	High	
	**Consensus**	**Consensus**	**Consensus**	No consensus	No consensus	
**54- Parent/caregiver's dietary intentions**						
**Round 1**						
Mean score	7.4	7.3	6.8	5.5	5.8	6.6

Median score	7.5	8.0	7.0	6.0	6.0	
MADM	1.2	0.9	1.4	1.6	1.4	
Agreement	Moderate	High	Low	Low	Moderate	
	No consensus	No consensus	No consensus	No consensus	No consensus	
**Round 2**						
Mean score	7.2	7.2	6.2	6.4	5.8	6.6
Median score	7.0	7.0	6.0	7.0	6.0	
MADM	1.1	1.0	1.4	1.1	1.3	
Agreement	Moderate	High	Moderate	High	Moderate	
	No consensus	No consensus	No consensus	No consensus	No consensus	
**Round 3**						
Mean score	7.4	7.6	7.6	6.7	5.9	7.0
Median score	8.0	8.0	8.0	7.0	6.0	
MADM	1.3	1.0	1.2	1.0	1.2	
Agreement	Moderate	High	Moderate	High	Moderate	
	No consensus	No consensus	No consensus	No consensus	No consensus	
**55- Parent/caregiver food variety**						
**Round 1**						
Mean score	7.3	7.3	**7.2**	6.3	6.3	6.9
Median score	7.0	8.0	**7.0**	6.0	6.0	
MADM	1.2	1.1	**1.1**	1.4	1.1	
Agreement	Moderate	Moderate	**High**	Moderate	Moderate	
	No consensus	No consensus	**Consensus**	No consensus	No consensus	
**Round 2**						
Mean score	**7.4**	**7.8**	6.9	6.6	6.3	7.0
Median score	**7.0**	**8.0**	7.0	7.0	6.0	
MADM	**1.0**	**1.0**	1.0	1.0	1.2	
Agreement	**High**	**High**	High	High	Moderate	
	**Consensus**	**Consensus**	No consensus	No consensus	No consensus	

**Round 3**						
Mean score	7.5	**8.1**	7.4	6.7	6.6	7.3
Median score	7.0	**8.0**	7.0	7.0	7.0	
MADM	1.0	**0.7**	1.2	0.8	1.1	
Agreement	High	**High**	Moderate	High	Moderate	
	No consensus	**Consensus**	No consensus	No consensus	No consensus	
**56- Parent/caregiver anxiety symptoms**						
**Round 1**						
Mean score	6.7	6.6	5.4	5.3	5.9	6.0
Median score	7.0	7.0	6.0	5.5	6.0	
MADM	1.3	1.5	2.0	1.7	1.4	
Agreement	Moderate	Low	Low	Low	Moderate	
	No consensus	No consensus	No consensus	No consensus	No consensus	
**Round 2**						
Mean score	6.8	7.1	6.1	5.8	5.5	6.3
Median score	7.0	7.5	6.0	6.0	6.0	
MADM	1.0	1.1	1.5	1.3	1.4	
Agreement	High	Moderate	Low	Moderate	Moderate	
	No consensus	No consensus	No consensus	No consensus	No consensus	
**Round 3**						
Mean score	6.7	8.0	6.8	6.4	6.0	6.8
Median score	7.0	8.0	7.0	7.0	6.0	
MADM	1.2	0.9	1.4	0.9	1.1	
Agreement	Moderate	High	Moderate	High	High	
	No consensus	No consensus	No consensus	No consensus	No consensus	
**57- Parent/caregiver concerns about child's weight**						
**Round 1**						
Mean score	6.9	7.1	6.1	6.5	6.2	6.6
Median score	7.0	8.0	6.0	7.0	7.0	7.0

MADM	1.1	1.2	1.1	1.4	1.3	
Agreement	Moderate	Moderate	Moderate	Low	Moderate	
	No consensus	No consensus	No consensus	No consensus	No consensus	
**Round 2**						
Mean score	6.6	7.1	6.4	6.9	6.2	6.6
Median score	7.0	7.0	6.0	7.0	7.0	
MADM	0.9	0.9	0.7	0.9	1.2	
Agreement	High	High	High	High	Moderate	
	No consensus	No consensus	No consensus	No consensus	No consensus	
**Round 3**						
Mean score	6.7	**7.4**	6.4	7.0	6.4	6.8
Median score	7.0	**8.0**	6.0	7.0	7.0	
MADM	1.2	**0.9**	0.8	0.9	0.9	
Agreement	Moderate	**High**	High	High	High	
	No consensus	**Consensus**	No consensus	No consensus	No consensus	
**58- Parent/caregiver depression symptoms**						
**Round 1**						
Mean score	6.7	6.9	5.3	5.4	6.1	6.1
Median score	7.0	8.0	5.0	5.0	6.0	
MADM	1.2	1.6	1.8	1.7	1.4	
Agreement	Moderate	Low	Low	Low	Moderate	
	No consensus	No consensus	No consensus	No consensus	No consensus	
**Round 2**						
Mean score	6.8	7.1	5.6	5.5	5.9	6.2
Median score	7.0	7.5	5.0	5.0	6.0	
MADM	1.1	1.4	1.2	1.3	1.3	
Agreement	High	Moderate	Moderate	Moderate	Moderate	
	No consensus	No consensus	No consensus	No consensus	No consensus	
**Round 3**						

Mean score	6.9	7.8	7.0	6.1	6.4	6.9
Median score	7.0	8.0	8.0	6.0	7.0	
MADM	1.0	0.8	1.0	0.9	1.0	
Agreement	High	High	High	High	High	
	No consensus	No consensus	No consensus	No consensus	No consensus	
**59- Parent/caregiver self-efficacy**						
**Round 1**						
Mean score	6.9	6.7	6.3	6.2	6.3	6.5
Median score	7.0	7.0	6.0	6.5	6.0	
MADM	1.0	1.3	1.3	1.9	1.1	
Agreement	High	Moderate	Moderate	Low	Moderate	
	No consensus	No consensus	No consensus	No consensus	No consensus	
**Round 2**						
Mean score	6.7	7.1	5.9	6.5	6.5	6.5
Median score	7.0	7.0	6.0	7.0	6.0	
MADM	0.9	1.0	1.0	1.3	1.1	
Agreement	High	High	High	Moderate	Moderate	
	No consensus	No consensus	No consensus	No consensus	No consensus	
**Round 3**						
Mean score	6.7	**7.6**	6.8	6.9	6.8	6.9
Median score	7.0	**8.0**	8.0	7.0	7.0	
MADM	0.9	**0.7**	1.2	1.1	0.9	
Agreement	High	**High**	Moderate	Moderate	High	
	No consensus	**Consensus**	No consensus	No consensus	No consensus	
**60- Parent/caregiver social support**						
**Round 1**						
Mean score	6.9	6.9	6.3	6.2	6.3	6.5
Median score	7.0	7.0	7.0	6.5	6.0	
MADM	1.3	1.0	1.6	2.0	1.3	

Agreement	Moderate	High	Low	Low	Moderate	
	No consensus	No consensus	No consensus	No consensus	No consensus	
**Round 2**						
Mean score	6.6	6.9	6.4	6.8	6.5	6.6
Median score	7.0	7.0	6.5	7.0	6.5	
MADM	1.1	0.9	1.6	1.5	1.3	
Agreement	Moderate	High	Low	Low	Moderate	
	No consensus	No consensus	No consensus	No consensus	No consensus	
**Round 3**						
Mean score	6.7	7.1	**7.6**	7.5	6.7	7.1
Median score	7.0	7.0	**8.0**	8.0	7.0	
MADM	1.1	0.7	**0.8**	1.0	1.1	
Agreement	Moderate	High	**High**	High	High	
	No consensus	No consensus	**Consensus**	No consensus	No consensus	
**61- Parent/caregiver stress**						
**Round 1**						
Mean score	6.8	6.9	5.8	5.8	6.2	6.3
Median score	7.0	7.0	6.0	6.0	6.0	
MADM	1.3	1.2	1.8	1.9	1.3	
Agreement	Moderate	Moderate	Low	Low	Moderate	
	No consensus	No consensus	No consensus	No consensus	No consensus	
**Round 2**						
Mean score	6.5	7.1	5.8	6.2	6.3	6.4
Median score	7.0	7.0	5.0	6.0	6.0	
MADM	1.1	0.8	1.4	1.3	1.2	
Agreement	Moderate	High	Moderate	Moderate	Moderate	
	No consensus	No consensus	No consensus	No consensus	No consensus	
**Round 3**						
Mean score	6.8	7.4	7.3	6.8	6.6	7.0

Median score	7.0	8.0	7.5	7.0	7.0	
MADM	0.9	1.0	1.3	1.0	0.9	
Agreement	High	High	Moderate	High	High	
	No consensus	No consensus	No consensus	No consensus	No consensus	
**62- Parent/caregiver attitudes**						
**Round 1**						
Mean score	**7.5**	7.0	6.2	6.2	6.2	6.6
Median score	**8.0**	8.0	6.0	7.0	6.0	
MADM	**1.1**	1.3	1.6	1.5	1.2	
Agreement	**Moderate**	Moderate	Low	Low	Moderate	
	**Consensus**	No consensus	No consensus	No consensus	No consensus	
**Round 2**						
Mean score	**7.5**	**7.4**	6.6	6.4	6.1	6.8
Median score	**8.0**	**7.5**	7.0	7.0	6.0	
MADM	**1.0**	**1.0**	1.1	1.1	1.1	
Agreement	**High**	**High**	Moderate	Moderate	Moderate	
	**Consensus**	**Consensus**	No consensus	No consensus	No consensus	
**Round 3**						
Mean score	**7.6**	**8.0**	7.8	6.9	6.4	7.3
Median score	**8.0**	**8.0**	9.0	7.0	6.0	
MADM	**1.1**	**0.7**	1.2	1.0	1.1	
Agreement	**High**	**High**	Moderate	High	Moderate	
	**Consensus**	**Consensus**	No consensus	No consensus	No consensus	
**63- Parent/caregiver awareness of behaviours**						
**Round 1**						
Mean score	7.3	6.8	6.6	6.4	6.2	6.7
Median score	8.0	7.0	7.0	6.0	6.0	
MADM	1.3	1.3	1.3	1.7	1.3	
Agreement	Moderate	Moderate	Moderate	Low	Moderate	

	No consensus	No consensus	No consensus	No consensus	No consensus	
**Round 2**						
Mean score	7.1	**7.1**	6.6	6.4	6.3	6.7
Median score	7.0	**7.0**	7.0	7.0	6.0	
MADM	1.1	**0.9**	1.2	1.4	1.2	
Agreement	Moderate	**High**	Moderate	Moderate	Moderate	
	No consensus	**Consensus**	No consensus	No consensus	No consensus	
**Round 3**						
Mean score	**7.4**	**7.7**	7.6	6.6	6.4	7.1
Median score	**7.0**	**7.5**	8.0	7.0	6.0	
MADM	**1.1**	**0.7**	1.2	1.1	1.1	
Agreement	**High**	**High**	Moderate	Moderate	Moderate	
	**Consensus**	**Consensus**	No consensus	No consensus	No consensus	
**64- Parent/caregiver beliefs**						
**Round 1**						
Mean score	7.2	6.9	6.6	6.2	6.2	6.6
Median score	7.0	7.5	6.0	7.0	6.5	
MADM	1.2	1.2	1.2	1.8	1.4	
Agreement	Moderate	Moderate	Moderate	Low	Moderate	
	No consensus	No consensus	No consensus	No consensus	No consensus	
**Round 2**						
Mean score	**7.2**	7.2	7.0	6.1	6.1	6.7
Median score	**7.0**	8.0	7.0	6.5	6.0	
MADM	**1.0**	1.0	0.6	1.5	1.2	
Agreement	**High**	High	High	Low	Moderate	
	**Consensus**	No consensus	No consensus	No consensus	No consensus	
**Round 3**						
Mean score	**7.5**	**7.7**	**7.8**	6.8	6.3	7.2
Median score	**7.0**	**8.0**	**8.0**	7.0	6.0	

MADM	**0.9**	**0.7**	**1.0**	1.1	1.1	
Agreement	**High**	**High**	**High**	Moderate	Moderate	
	**Consensus**	**Consensus**	**Consensus**	No consensus	No consensus	
**65- Parent/caregiver health literacy**						
**Round 1**						
Mean score	7.5	7.0	**7.2**	6.7	6.6	7.0
Median score	8.0	7.0	**8.0**	7.0	7.0	
MADM	1.2	1.3	**1.1**	1.4	1.3	
Agreement	Moderate	Moderate	**Moderate**	Low	Moderate	
	No consensus	No consensus	**Consensus**	No consensus	No consensus	
**Round 2**						
Mean score	**7.5**	**7.5**	7.1	7.2	6.7	7.2
Median score	**8.0**	**7.5**	7.0	7.5	7.0	
MADM	**1.0**	**1.1**	0.7	1.4	1.1	
Agreement	**High**	**Moderate**	High	Moderate	Moderate	
	**Consensus**	**Consensus**	No consensus	No consensus	No consensus	
**Round 3**						
Mean score	**7.7**	**7.8**	**7.2**	7.3	6.9	7.4
Median score	**8.0**	**8.0**	**7.0**	8.0	7.0	
MADM	**0.9**	**0.5**	**0.6**	1.1	1.0	
Agreement	**High**	**High**	**High**	Moderate	High	
	**Consensus**	**Consensus**	**Consensus**	No consensus	No consensus	
**66- Parent/caregiver knowledge**						
**Round 1**						
Mean score	7.6	7.1	7.1	6.8	6.3	7.0
Median score	8.0	7.0	7.0	7.0	6.0	
MADM	1.1	1.2	1.3	1.3	1.2	
Agreement	Moderate	Moderate	Moderate	Moderate	Moderate	
	No consensus	No consensus	No consensus	No consensus	No consensus	

**Round 2**						
Mean score	**7.6**	**7.6**	6.8	7.0	6.2	7.0
Median score	**7.0**	**7.0**	6.5	7.0	6.0	
MADM	**1.0**	**1.0**	1.0	1.1	1.1	
Agreement	**High**	**High**	High	Moderate	Moderate	
	**Consensus**	**Consensus**	No consensus	No consensus	No consensus	
**Round 3**						
Mean score	**7.6**	**7.7**	7.2	7.1	6.1	7.1
Median score	**8.0**	**7.0**	7.0	7.0	6.0	
MADM	**0.9**	**0.7**	1.0	1.2	1.0	
Agreement	**High**	**High**	High	Moderate	High	
	**Consensus**	**Consensus**	No consensus	No consensus	No consensus	
**67- Parent/caregiver readiness to change**						
**Round 1**						
Mean score	**8.0**	7.2	7.2	6.4	6.5	7.0
Median score	**9.0**	7.0	7.0	6.0	6.0	
MADM	**1.0**	1.2	1.0	1.7	1.3	
Agreement	**Moderate**	Moderate	High	Low	Moderate	
	**Consensus**	No consensus	No consensus	No consensus	No consensus	
**Round 2**						
Mean score	**8.1**	**7.7**	7.3	6.9	6.5	7.3
Median score	**9.0**	**8.0**	7.0	7.5	6.0	
MADM	**0.9**	**0.9**	1.3	1.6	1.2	
Agreement	**High**	**High**	Moderate	Low	Moderate	
	**Consensus**	**Consensus**	No consensus	No consensus	No consensus	
**Round 3**						
Mean score	**7.9**	**8.2**	7.2	6.9	6.7	7.4
Median score	**9.0**	**8.0**	7.0	7.0	6.5	
MADM	**1.1**	**0.5**	1.0	1.4	1.3	

Agreement	**Moderate**	**High**	High	Low	Moderate	
	**Consensus**	**Consensus**	No consensus	No consensus	No consensus	
**68- Parenting style**						
**Round 1**						
Mean score	6.6	6.6	6.6	6.1	6.1	6.4
Median score	7.0	7.0	7.0	7.0	6.0	
MADM	1.2	1.4	1.6	1.8	1.4	
Agreement	Moderate	Low	Low	Low	Moderate	
	No consensus	No consensus	No consensus	No consensus	No consensus	
**Round 2**						
Mean score	6.9	7.2	6.1	6.1	6.2	6.5
Median score	7.0	8.0	6.0	7.0	6.0	
MADM	0.9	1.2	1.1	1.3	1.2	
Agreement	High	Moderate	Moderate	Moderate	Moderate	
	No consensus	No consensus	No consensus	No consensus	No consensus	
**Round 3**						
Mean score	**7.1**	**7.8**	6.4	6.8	6.3	6.9
Median score	**7.0**	**8.0**	6.0	7.0	6.0	
MADM	**0.9**	**0.5**	1.2	0.9	1.3	
Agreement	**High**	**High**	Moderate	High	Moderate	
	**Consensus**	**Consensus**	No consensus	No consensus	No consensus	
**69- Parent/caregiver responsiveness**						
**Round 1**						
Mean score	7.2	6.7	6.5	6.2	6.4	6.6
Median score	7.0	7.0	7.0	6.0	6.0	
MADM	1.0	1.2	1.4	1.9	1.3	
Agreement	High	Moderate	Low	Low	Moderate	
	No consensus	No consensus	No consensus	No consensus	No consensus	
**Round 2**						

Mean score	7.3	6.9	6.1	6.6	6.4	6.7
Median score	7.0	7.0	6.0	7.0	6.0	
MADM	0.8	0.9	0.9	1.1	1.2	
Agreement	High	High	High	High	Moderate	
	No consensus	No consensus	No consensus	No consensus	No consensus	
**Round 3**						
Mean score	**7.7**	**7.3**	7.0	6.6	6.6	7.0
Median score	**8.0**	**7.5**	8.0	7.0	6.0	
MADM	**0.9**	**0.7**	1.0	1.1	1.1	
Agreement	**High**	**High**	High	Moderate	Moderate	
	**Consensus**	**Consensus**	No consensus	No consensus	No consensus	
**70- Family functioning**						
**Round 1**						
Mean score	7.0	6.4	6.7	5.9	6.4	6.5
Median score	7.0	6.5	6.5	6.0	6.0	
MADM	1.2	1.3	1.2	1.7	1.3	
Agreement	Moderate	Moderate	Moderate	Low	Moderate	
	No consensus	No consensus	No consensus	No consensus	No consensus	
**Round 2**						
Mean score	6.8	7.0	6.4	6.2	6.4	6.6
Median score	7.0	7.5	6.0	7.0	6.0	
MADM	0.8	1.0	0.9	1.2	1.1	
Agreement	High	High	High	Moderate	Moderate	
	No consensus	No consensus	No consensus	No consensus	No consensus	
**Round 3**						
Mean score	7.1	**7.3**	7.2	6.6	6.5	6.9
Median score	7.0	**8.0**	8.0	7.0	6.0	
MADM	0.9	**0.7**	0.8	0.9	1.0	
Agreement	High	**High**	High	High	High	

	No consensus	**Consensus**	No consensus	No consensus	No consensus	
**71- Parent/caregiver perception of child weight**						
**Round 1**						
Mean score	7.3	7.1	6.8	6.8	6.4	6.9
Median score	7.0	7.0	7.0	7.0	7.0	
MADM	1.0	1.1	1.1	1.5	1.3	
Agreement	High	Moderate	Moderate	Low	Moderate	
	No consensus	No consensus	No consensus	No consensus	No consensus	
**Round 2**						
Mean score	**7.2**	6.9	6.8	6.8	6.4	6.8
Median score	**7.0**	7.0	7.0	7.0	7.0	
MADM	**0.9**	0.7	1.0	0.9	1.0	
Agreement	**High**	High	High	High	High	
	**Consensus**	No consensus	No consensus	No consensus	No consensus	
**Round 3**						
Mean score	7.1	**7.2**	7.0	6.8	6.6	6.9
Median score	7.0	**7.0**	8.0	7.0	7.0	
MADM	1.2	**0.8**	1.4	0.9	1.0	
Agreement	Moderate	**High**	Moderate	High	High	
	No consensus	**Consensus**	No consensus	No consensus	No consensus	
**72- Perception of parent/caregiver influence on child's health behaviours**						
**Round 1**						
Mean score	7.4	7.0	7.1	6.7	6.4	6.9
Median score	7.0	7.0	7.5	7.0	7.0	
MADM	1.1	1.1	1.2	1.7	1.3	
Agreement	High	High	Moderate	Low	Moderate	
	No consensus	No consensus	No consensus	No consensus	No consensus	
**Round 2**						
Mean score	**7.5**	**7.5**	7.0	6.8	6.4	7.0

Median score	**7.5**	**7.0**	7.0	7.0	7.0	
MADM	**1.0**	**0.7**	1.1	1.5	1.3	
Agreement	**High**	**High**	Moderate	Low	Moderate	
	**Consensus**	**Consensus**	No consensus	No consensus	No consensus	
**Round 3**						
Mean score	**7.5**	**8.0**	7.0	7.1	6.5	7.2
Median score	**8.0**	**8.0**	7.0	7.0	7.0	
MADM	**0.7**	**0.7**	1.2	1.2	1.1	
Agreement	**High**	**High**	Moderate	Moderate	Moderate	
	**Consensus**	**Consensus**	No consensus	No consensus	No consensus	
**73- Parent/caregiver perceptions of adequacy of resources and barriers**						
**Round 1**						
Mean score	7.4	7.1	6.6	6.9	6.5	6.9
Median score	8.0	7.5	7.0	7.0	7.0	
MADM	1.1	1.3	1.4	1.4	1.2	
Agreement	Moderate	Moderate	Low	Moderate	Moderate	
	No consensus	No consensus	No consensus	No consensus	No consensus	
**Round 2**						
Mean score	**7.4**	**7.4**	6.9	**7.1**	6.6	7.1
Median score	**8.0**	**8.0**	7.0	**7.0**	7.0	
MADM	**0.7**	**1.0**	1.1	**0.9**	1.1	
Agreement	**High**	**High**	Moderate	**High**	Moderate	
	**Consensus**	**Consensus**	No consensus	**Consensus**	No consensus	
**Round 3**						
Mean score	**7.4**	**7.5**	6.8	**7.6**	6.7	7.2
Median score	**8.0**	**7.0**	7.0	**7.0**	7.0	
MADM	**0.6**	**0.5**	1.0	**0.6**	1.0	
Agreement	**High**	**High**	High	**High**	High	
	**Consensus**	**Consensus**	No consensus	**Consensus**	No consensus	

**74- Parent/caregiver feeding control**						
**Round 1**						
Mean score	**7.8**	6.7	6.3	6.7	6.6	6.8
Median score	**8.0**	7.0	7.0	7.0	7.0	
MADM	**1.0**	1.6	1.4	1.4	1.3	
Agreement	**High**	Low	Moderate	Low	Moderate	
	**Consensus**	No consensus	No consensus	No consensus	No consensus	
**Round 2**						
Mean score	**7.8**	**7.8**	6.2	6.8	6.7	7.1
Median score	**8.0**	**8.0**	7.0	7.0	7.0	
MADM	**0.7**	**0.8**	1.3	1.2	1.1	
Agreement	**High**	**High**	Moderate	Moderate	Moderate	
	**Consensus**	**Consensus**	No consensus	No consensus	No consensus	
**Round 3**						
Mean score	**8.0**	**8.0**	4.8	7.1	6.8	6.9
Median score	**8.0**	**8.0**	5.5	7.0	7.0	
MADM	**0.9**	**0.9**	2.2	1.2	1.1	
Agreement	**High**	**High**	Low	Moderate	Moderate	
	**Consensus**	**Consensus**	No consensus	No consensus	No consensus	
**75- Parent/caregiver emotional feeding**						
**Round 1**						
Mean score	**7.8**	6.8	6.6	6.3	6.4	6.8
Median score	**8.0**	7.0	7.0	6.0	6.0	
MADM	**0.9**	1.6	1.3	1.5	1.4	
Agreement	**High**	Low	Moderate	Low	Moderate	
	**Consensus**	No consensus	No consensus	No consensus	No consensus	
**Round 2**						
Mean score	**7.6**	7.7	6.9	6.3	6.6	7.0
Median score	**8.0**	8.0	7.0	6.0	7.0	

MADM	**0.9**	0.8	1.1	1.3	1.3	
Agreement	**High**	High	Moderate	Moderate	Moderate	
	**Consensus**	No consensus	No consensus	No consensus	No consensus	
**Round 3**						
Mean score	**7.7**	**8.0**	5.7	6.8	6.7	7.0
Median score	**8.0**	**8.0**	6.0	7.0	7.0	
MADM	**0.8**	**0.6**	1.3	1.3	1.3	
Agreement	**High**	**High**	Moderate	Moderate	Moderate	
	**Consensus**	**Consensus**	No consensus	No consensus	No consensus	
**76- Parent/caregiver feeding encouragement**						
**Round 1**						
Mean score	7.3	**7.4**	6.5	6.4	6.3	6.8
Median score	7.0	**7.5**	7.0	6.0	6.0	
MADM	1.0	**1.3**	1.1	1.5	1.2	
Agreement	High	**Moderate**	Moderate	Low	Moderate	
	No consensus	**Consensus**	No consensus	No consensus	No consensus	
**Round 2**						
Mean score	7.1	**7.9**	6.5	6.4	6.4	6.9
Median score	7.0	**8.0**	7.0	7.0	6.0	
MADM	0.9	**0.7**	1.0	1.1	1.0	
Agreement	High	**High**	High	Moderate	High	
	No consensus	**Consensus**	No consensus	No consensus	No consensus	
**Round 3**						
Mean score	7.1	**7.9**	6.8	6.8	6.5	7.0
Median score	7.0	**8.0**	6.5	7.0	7.0	
MADM	1.2	**0.7**	1.2	1.1	1.0	
Agreement	Moderate	**High**	Moderate	Moderate	High	
	No consensus	**Consensus**	No consensus	No consensus	No consensus	
**77- Parent/caregiver feeding interaction**						

**Round 1**						
Mean score	7.7	7.3	6.4	6.9	6.7	7.0
Median score	8.0	7.5	7.0	7.0	7.0	
MADM	0.9	1.1	1.5	1.3	1.3	
Agreement	High	Moderate	Low	Moderate	Moderate	
	No consensus	No consensus	No consensus	No consensus	No consensus	
**Round 2**						
Mean score	7.4	**7.8**	6.8	6.9	6.7	7.1
Median score	7.0	**8.0**	6.5	7.0	7.0	
MADM	1.1	**0.8**	1.3	1.0	1.2	
Agreement	Moderate	**High**	Moderate	High	Moderate	
	No consensus	**Consensus**	No consensus	No consensus	No consensus	
**Round 3**						
Mean score	**7.7**	**7.9**	7.0	7.0	6.7	7.3
Median score	**8.0**	**8.0**	7.0	7.0	7.0	
MADM	**1.1**	**0.7**	1.7	1.3	1.3	
Agreement	**Moderate**	**High**	Low	Moderate	Moderate	
	**Consensus**	**Consensus**	No consensus	No consensus	No consensus	
**78- Parent/caregiver feeding structure**						
**Round 1**						
Mean score	7.5	7.0	6.7	6.7	6.4	6.9
Median score	8.0	7.5	7.0	6.0	6.0	
MADM	1.1	1.3	1.4	1.4	1.3	
Agreement	Moderate	Moderate	Moderate	Moderate	Moderate	
	No consensus	No consensus	No consensus	No consensus	No consensus	
**Round 2**						
Mean score	7.4	**7.8**	6.5	6.6	6.4	6.9
Median score	8.0	**8.0**	6.0	7.0	6.0	
MADM	1.1	**0.6**	1.2	1.0	1.0	

Agreement	High	**High**	Moderate	High	High	
	No consensus	**Consensus**	No consensus	No consensus	No consensus	
**Round 3**						
Mean score	7.3	**7.9**	6.2	6.8	6.5	6.9
Median score	7.0	**8.0**	5.5	7.0	6.0	
MADM	1.3	**0.7**	1.2	1.3	1.1	
Agreement	Moderate	**High**	Moderate	Moderate	Moderate	
	No consensus	**Consensus**	No consensus	No consensus	No consensus	
**79- Parent/caregiver feeding style**						
**Round 1**						
Mean score	7.3	6.6	6.3	6.4	6.5	6.6
Median score	8.0	7.0	7.0	7.0	7.0	
MADM	1.2	1.4	1.4	1.2	1.2	
Agreement	Moderate	Low	Low	Moderate	Moderate	
	No consensus	No consensus	No consensus	No consensus	No consensus	
**Round 2**						
Mean score	7.1	**7.7**	6.3	6.7	6.5	6.9
Median score	7.0	**8.0**	6.0	7.0	7.0	
MADM	1.0	**0.8**	1.0	0.9	1.1	
Agreement	High	**High**	High	High	Moderate	
	No consensus	**Consensus**	No consensus	No consensus	No consensus	
**Round 3**						
Mean score	7.4	**8.0**	6.3	6.9	6.4	7.0
Median score	7.0	**8.0**	6.5	7.0	7.0	
MADM	1.2	**0.6**	1.7	1.2	1.2	
Agreement	Moderate	**High**	Low	Moderate	Moderate	
	No consensus	**Consensus**	No consensus	No consensus	No consensus	
**80- Parent/caregiver using food as a reward**						
**Round 1**						

Mean score	**7.7**	6.6	6.8	6.8	6.6	6.9
Median score	**8.0**	7.0	7.0	7.0	7.0	
MADM	**1.2**	1.7	1.5	1.6	1.2	
Agreement	**Moderate**	Low	Low	Low	Moderate	
	**Consensus**	No consensus	No consensus	No consensus	No consensus	
**Round 2**						
Mean score	**7.9**	**7.9**	6.6	7.0	7.0	7.3
Median score	**8.0**	**8.0**	7.0	7.0	7.0	
MADM	**0.9**	**0.8**	1.3	1.3	0.8	
Agreement	**High**	**High**	Moderate	Moderate	High	
	**Consensus**	**Consensus**	No consensus	No consensus	No consensus	
**Round 3**						
Mean score	**8.1**	**8.0**	6.7	7.2	7.0	7.4
Median score	**8.0**	**8.0**	6.5	8.0	7.0	
MADM	**0.7**	**0.9**	1.0	1.4	0.9	
Agreement	**High**	**High**	High	Low	High	
	**Consensus**	**Consensus**	No consensus	No consensus	No consensus	
**81- Parent/caregiver frequency of bottle use**						
**Round 1**						
Mean score	7.8	6.6	6.1	6.4	6.0	6.6
Median score	8.0	7.0	6.0	7.0	6.0	
MADM	1.0	1.4	1.9	1.9	1.2	
Agreement	Moderate	Moderate	Low	Low	Moderate	
	No consensus	No consensus	No consensus	No consensus	No consensus	
**Round 2**						
Mean score	**7.79**	7.38	5.64	6.73	6.02	6.7
Median score	**8.00**	8.00	6.00	7.00	6.00	
MADM	**1.10**	0.88	0.91	1.47	1.02	
Agreement	**Moderate**	High	High	Low	High	

	**Consensus**	No consensus	No consensus	No consensus	No consensus	
**Round 3**						
Mean score	**7.9**	7.2	5.4	6.7	6.1	6.7
Median score	**8.0**	7.5	5.0	7.0	6.0	
MADM	**1.0**	1.2	1.6	1.7	1.1	
Agreement	**High**	Moderate	Low	Low	Moderate	
	**Consensus**	No consensus	No consensus	No consensus	No consensus	
**82- Parent/caregiver promoting autonomy**						
**Round 1**						
Mean score	**7.5**	6.8	6.8	6.3	6.4	6.8
Median score	**8.0**	6.0	7.0	6.0	6.0	
MADM	**1.1**	1.3	1.3	1.5	1.2	
Agreement	**Moderate**	Moderate	Moderate	Low	Moderate	
	**Consensus**	No consensus	No consensus	No consensus	No consensus	
**Round 2**						
Mean score	**7.5**	7.8	**7.1**	6.3	6.6	7.0
Median score	**7.5**	8.0	**7.0**	6.5	7.0	
MADM	**1.0**	0.7	**1.3**	1.3	1.0	
Agreement	**High**	High	**Moderate**	Moderate	High	
	**Consensus**	No consensus	**Consensus**	No consensus	No consensus	
**Round 3**						
Mean score	7.5	**8.0**	**7.3**	6.7	6.7	7.3
Median score	8.0	**8.0**	**7.5**	7.0	7.0	
MADM	1.0	**0.6**	**1.3**	1.2	1.0	
Agreement	High	**High**	**Moderate**	Moderate	High	
	No consensus	**Consensus**	**Consensus**	No consensus	No consensus	
**83- Parent/caregiver that is responsible for feeding**						
**Round 1**						
Mean score	7.1	6.7	6.1	6.9	6.2	6.6

Median score	7.0	7.0	6.5	7.0	6.0	
MADM	1.5	1.5	1.9	1.5	1.4	
Agreement	Low	Low	Low	Low	Low	
	No consensus	No consensus	No consensus	No consensus	No consensus	
**Round 2**						
Mean score	**7.5**	7.7	5.4	6.9	6.2	6.7
Median score	**7.5**	8.0	5.0	7.0	6.0	
MADM	**1.1**	0.8	1.5	1.3	1.3	
Agreement	**Moderate**	High	Low	Moderate	Moderate	
	**Consensus**	No consensus	No consensus	No consensus	No consensus	
**Round 3**						
Mean score	**7.5**	**8.0**	6.0	7.1	6.0	6.9
Median score	**7.0**	**8.0**	6.5	7.0	6.0	
MADM	**1.1**	**0.6**	1.7	1.2	1.3	
Agreement	**Moderate**	**High**	Low	Moderate	Moderate	
	**Consensus**	**Consensus**	No consensus	No consensus	No consensus	
**84- Food environment**						
**Round 1**						
Mean score	7.6	6.5	7.2	8.4	7.4	7.4
Median score	8	7	7	9	7	
MADM	1.1	1.3	1.2	0.6	1.1	
Agreement	High	Moderate	Moderate	High	Moderate	
	Consensus	No consensus	Consensus	Consensus	Consensus	
**Round 2**						
Mean score	7.9	7.3	7.4	8.4	7.7	7.7
Median score	8	7	8	9	8	
MADM	0.9	1.1	0.8	0.6	1.0	
Agreement	High	Moderate	High	High	High	
	Consensus	Consensus	No consensus	Consensus	Consensus	

**Round 3**						
Mean score	7.8	8.0	7.8	8.7	7.8	8.0
Median score	8.0	8.0	8.0	9.0	8.0	
MADM	0.9	0.9	0.2	0.3	0.9	
Agreement	High	High	High	High	High	
	Consensus	Consensus	Consensus	Consensus	Consensus	
**85- Household food security**						
**Round 1**						
Mean score	8.0	7.5	7.9	8.2	7.6	7.8
Median score	8	8	9	9	7	
MADM	1.0	0.9	1.1	0.9	1.1	
Agreement	High	High	Moderate	High	High	
	Consensus	Consensus	Consensus	Consensus	Consensus	
**Round 2**						
Mean score	8.3	7.8	7.7	8.5	7.7	8.0
Median score	9	9	8	9	8	
MADM	0.7	1.2	1.2	0.5	1.1	
Agreement	High	Moderate	Moderate	High	High	
	Consensus	Consensus	Consensus	Consensus	Consensus	
**Round 3**						
Mean score	8.5	8.3	8.0	8.5	7.9	8.2
Median score	9	9	8	9	8	
MADM	0.5	0.7	0.7	0.5	1.0	
Agreement	High	High	High	High	High	
	Consensus	Consensus	Consensus	Consensus	Consensus	
**86- Family meal environment**						
**Round 1**						
Mean score	**7.8**	6.9	7.1	**7.7**	**7.3**	7.4
Median score	**8.0**	7.0	7.0	**8.0**	**7.0**	

MADM	**1.0**	1.3	1.4	**0.9**	**1.0**	
Agreement	**High**	Moderate	Moderate	**High**	**High**	
	**Consensus**	No consensus	No consensus	**Consensus**	**Consensus**	
**Round 2**						
Mean score	**8.1**	**7.8**	**7.2**	7.6	**7.4**	7.6
Median score	**8.0**	**8.0**	**7.0**	8.0	**7.0**	
MADM	**0.8**	**1.0**	**0.8**	1.0	**0.9**	
Agreement	**High**	**High**	**High**	High	**High**	
	**Consensus**	**Consensus**	**Consensus**	No consensus	**Consensus**	
**Round 3**						
Mean score	**8.0**	**8.3**	**8.0**	7.7	**7.6**	7.9
Median score	**8.0**	**8.5**	**8.0**	7.0	**7.0**	
MADM	**0.9**	**0.7**	**0.7**	1.1	**0.9**	
Agreement	**High**	**High**	**High**	Moderate	**High**	
	**Consensus**	**Consensus**	**Consensus**	No consensus	**Consensus**	
**87- Early childhood education and care (ECEC) environment**						
**Round 1**						
Mean score	7.2	7.3	7.2	**7.9**	7.0	7.3
Median score	7.0	7.0	7.0	**8.0**	7.0	
MADM	1.3	1.2	1.3	**0.8**	1.1	
Agreement	Moderate	Moderate	Moderate	**High**	High	
	No consensus	No consensus	No consensus	**Consensus**	No consensus	
**Round 2**						
Mean score	7.4	**7.7**	**7.5**	**8.0**	7.1	7.5
Median score	7.0	**7.5**	**7.0**	**8.0**	7.0	
MADM	1.1	**0.7**	**1.0**	**0.7**	0.9	
Agreement	Moderate	**High**	**High**	**High**	High	
	No consensus	**Consensus**	**Consensus**	**Consensus**	No consensus	
**Round 3**						

Mean score	7.3	**8.0**	**8.2**	**8.1**	7.2	7.7
Median score	7.0	**8.0**	**8.5**	**8.5**	7.0	
MADM	1.1	**0.7**	**0.8**	**0.9**	0.8	
Agreement	High	**High**	**High**	**High**	High	
	No consensus	**Consensus**	**Consensus**	**Consensus**	No consensus	
**88- Healthcare environment**						
**Round 1**						
Mean score	6.4	6.6	6.4	6.3	6.0	6.3
Median score	6.0	7.0	7.0	6.0	6.0	
MADM	1.4	0.9	2.0	1.5	1.5	
Agreement	Moderate	High	Low	Low	Low	
	No consensus	No consensus	No consensus	No consensus	No consensus	
**Round 2**						
Mean score	6.8	7.0	6.1	5.9	5.9	6.3
Median score	7.0	7.0	6.5	5.5	6.0	
MADM	1.1	0.7	1.6	1.3	1.3	
Agreement	Moderate	High	Low	Moderate	Moderate	
	No consensus	No consensus	No consensus	No consensus	No consensus	
**Round 3**						
Mean score	6.6	**8.0**	6.3	6.4	5.8	6.6
Median score	6.0	**8.0**	7.0	7.0	6.0	
MADM	1.0	**0.7**	1.3	1.2	1.2	
Agreement	High	**High**	Moderate	Moderate	Moderate	
	No consensus	**Consensus**	No consensus	No consensus	No consensus	
**89- Child internalising and/or externalising behaviours**						
**Round 1**						
Mean score	6.3	6.6	6.3	5.4	5.6	6.0
Median score	6.0	6.5	7.0	6.0	6.0	
MADM	1.5	1.3	2.0	1.5	1.3	

Agreement	Low	Moderate	Low	Low	Moderate	
	No consensus	No consensus	No consensus	No consensus	No consensus	
**Round 2**						
Mean score	6.4	7.0	5.9	5.5	5.6	6.1
Median score	6.5	7.5	6.0	6.0	6.0	
MADM	1.1	1.2	1.4	0.9	1.0	
Agreement	High	Moderate	Moderate	High	High	
	No consensus	No consensus	No consensus	No consensus	No consensus	
**Round 3**						
Mean score	6.5	7.0	6.8	5.7	5.7	6.3
Median score	6.0	7.0	7.0	6.0	6.0	
MADM	0.9	0.7	1.4	1.0	0.8	
Agreement	High	High	Moderate	High	High	
	No consensus	No consensus	No consensus	No consensus	No consensus	
**90- Child emotion regulation**						
**Round 1**						
Mean score	6.7	6.8	6.7	5.8	6.1	6.4
Median score	7.0	7.0	7.0	6.0	6.0	
MADM	1.3	1.3	1.7	1.6	1.3	
Agreement	Moderate	Moderate	Low	Low	Moderate	
	No consensus	No consensus	No consensus	No consensus	No consensus	
**Round 2**						
Mean score	6.9	6.8	6.5	6.1	6.0	6.5
Median score	7.0	7.0	6.0	6.0	6.0	
MADM	1.2	1.2	1.2	1.3	1.2	
Agreement	Moderate	Moderate	Moderate	Moderate	Moderate	
	No consensus	No consensus	No consensus	No consensus	No consensus	
**Round 3**						
Mean score	6.9	6.7	6.8	6.5	6.0	6.6

Median score	7.0	7.0	7.0	6.5	6.0	
MADM	1.2	0.7	1.0	1.5	1.1	
Agreement	Moderate	High	High	Low	High	
	No consensus	No consensus	No consensus	No consensus	No consensus	
**91- Child emotional development**						
**Round 1**						
Mean score	6.4	6.8	6.9	5.5	5.7	6.3
Median score	6.0	8.0	7.0	6.0	6.0	
MADM	1.3	1.4	1.6	1.8	1.3	
Agreement	Moderate	Moderate	Low	Low	Moderate	
	No consensus	No consensus	No consensus	No consensus	No consensus	
**Round 2**						
Mean score	6.5	7.3	6.5	5.7	5.7	6.3
Median score	6.0	8.0	7.0	5.5	6.0	
MADM	1.3	0.9	1.2	1.3	1.2	
Agreement	Moderate	High	Moderate	Moderate	Moderate	
	No consensus	No consensus	No consensus	No consensus	No consensus	
**Round 3**						
Mean score	6.6	7.2	**7.7**	6.3	5.7	6.7
Median score	7.0	7.5	**8.0**	6.0	6.0	
MADM	1.2	1.2	**1.0**	1.3	1.1	
Agreement	Moderate	Moderate	**High**	Moderate	High	
	No consensus	No consensus	**Consensus**	No consensus	No consensus	
**92- Child school readiness**						
**Round 1**						
Mean score	5.5	6.0	5.7	5.2	5.2	5.5
Median score	6.0	6.0	6.0	5.0	5.0	
MADM	1.6	1.1	1.7	1.6	1.5	
Agreement	Low	Moderate	Low	Low	Low	

	No consensus	No consensus	No consensus	No consensus	No consensus	
**Round 2**						
Mean score	5.5	6.3	5.5	5.3	5.2	5.6
Median score	5.5	6.0	6.0	5.0	5.0	
MADM	1.5	0.9	1.2	1.0	1.5	
Agreement	Low	High	Moderate	High	Low	
	No consensus	No consensus	No consensus	No consensus	No consensus	
**Round 3**						
Mean score	5.4	6.3	6.5	5.6	5.2	5.8
Median score	6.0	6.0	6.5	5.0	5.0	
MADM	1.5	0.7	1.2	1.2	1.3	
Agreement	Low	High	Moderate	Moderate	Moderate	
	No consensus	No consensus	No consensus	No consensus	No consensus	
**93- Child wellbeing**						
**Round 1**						
Mean score	6.9	**7.6**	7.5	7.0	6.5	7.1
Median score	7.0	**8.0**	8.0	7.0	7.0	
MADM	1.4	**0.8**	1.2	1.5	1.3	
Agreement	Low	**High**	High	Low	Moderate	
	No consensus	**Consensus**	No consensus	No consensus	No consensus	
**Round 2**						
Mean score	**7.3**	**7.7**	**7.8**	**7.6**	6.8	7.5
Median score	**8.0**	**8.0**	**8.0**	**8.0**	7.0	
MADM	**1.0**	**0.6**	**0.7**	**0.9**	1.0	
Agreement	**High**	**High**	**High**	**High**	High	
	**Consensus**	**Consensus**	**Consensus**	**Consensus**	No consensus	
**Round 3**						
Mean score	**7.2**	**7.8**	**8.8**	**7.7**	6.9	7.7
Median score	**8.0**	**8.0**	**9.0**	**8.0**	7.0	

MADM	**0.9**	**0.5**	**0.2**	**0.9**	1.0	
Agreement	**High**	**High**	**High**	**High**	High	
	**Consensus**	**Consensus**	**Consensus**	**Consensus**	No consensus	
**94- Child attention control**						
**Round 1**						
Mean score	5.8	6.2	5.7	5.3	5.4	5.7
Median score	6.0	6.0	6.0	4.0	5.0	
MADM	1.6	1.2	1.7	1.7	1.3	
Agreement	Low	Moderate	Low	Low	Moderate	
	No consensus	No consensus	No consensus	No consensus	No consensus	
**Round 2**						
Mean score	6.2	6.1	5.4	5.2	5.2	5.6
Median score	6.0	6.0	6.0	4.0	5.0	
MADM	1.5	0.9	1.2	1.4	1.2	
Agreement	Low	High	Moderate	Moderate	Moderate	
	No consensus	No consensus	No consensus	No consensus	No consensus	
**Round 3**						
Mean score	6.5	6.2	6.0	5.4	5.1	5.8
Median score	7.0	6.0	6.0	5.0	5.0	
MADM	1.5	0.8	0.7	1.3	1.0	
Agreement	Low	High	High	Moderate	High	
	No consensus	No consensus	No consensus	No consensus	No consensus	
**95- Child cognitive development**						
**Round 1**						
Mean score	6.0	6.1	6.3	5.9	5.8	6.0
Median score	6.0	6.5	6.0	6.0	6.0	
MADM	1.5	1.6	1.7	2.0	1.3	
Agreement	Low	Low	Low	Low	Moderate	
	No consensus	No consensus	No consensus	No consensus	No consensus	

**Round 2**						
Mean score	6.3	6.7	6.2	5.6	5.7	6.1
Median score	6.0	7.5	6.0	6.0	6.0	
MADM	1.3	1.3	1.1	1.3	1.2	
Agreement	Moderate	Moderate	Moderate	Moderate	Moderate	
	No consensus	No consensus	No consensus	No consensus	No consensus	
**Round 3**						
Mean score	6.3	7.0	7.0	6.1	5.7	6.4
Median score	6.0	7.5	6.5	6.0	6.0	
MADM	1.5	1.0	1.0	1.1	1.1	
Agreement	Low	High	High	Moderate	Moderate	
	No consensus	No consensus	No consensus	No consensus	No consensus	
**96- Child executive function**						
**Round 1**						
Mean score	5.8	5.9	6.3	5.3	5.7	5.8
Median score	6.0	6.0	7.0	5.0	6.0	
MADM	1.5	1.2	1.7	1.8	1.4	
Agreement	Low	Moderate	Low	Low	Moderate	
	No consensus	No consensus	No consensus	No consensus	No consensus	
**Round 2**						
Mean score	6.1	6.5	6.1	5.4	5.6	5.9
Median score	6.0	7.0	7.0	6.0	6.0	
MADM	1.1	1.3	1.5	1.4	1.4	
Agreement	Moderate	Moderate	Low	Moderate	Moderate	
	No consensus	No consensus	No consensus	No consensus	No consensus	
**Round 3**						
Mean score	5.8	6.8	6.8	5.8	5.6	6.2
Median score	6.0	7.5	7.0	6.0	6.0	
MADM	1.4	1.2	0.8	1.3	1.2	

Agreement	Moderate	Moderate	High	Moderate	Moderate	
	No consensus	No consensus	No consensus	No consensus	No consensus	
**97- Child self-regulation**						
**Round 1**						
Mean score	6.5	6.8	6.9	5.9	6.3	6.5
Median score	7.0	7.0	7.0	6.0	7.0	
MADM	1.4	1.1	1.6	1.9	1.3	
Agreement	Low	High	Low	Low	Moderate	
	No consensus	No consensus	No consensus	No consensus	No consensus	
**Round 2**						
Mean score	6.8	7.2	6.7	6.5	6.4	6.7
Median score	7.0	7.5	6.0	7.0	7.0	
MADM	1.2	1.0	1.5	1.1	1.2	
Agreement	Moderate	High	Low	High	Moderate	
	No consensus	No consensus	No consensus	No consensus	No consensus	
**Round 3**						
Mean score	7.1	**7.2**	6.2	6.6	6.4	6.7
Median score	7.0	**7.0**	6.0	7.0	7.0	
MADM	1.2	**0.5**	1.2	1.1	1.1	
Agreement	Moderate	**High**	Moderate	Moderate	Moderate	
	No consensus	**Consensus**	No consensus	No consensus	No consensus	
**98- Child fundamental movement skills**						
**Round 1**						
Mean score	6.4	6.4	7.0	6.8	5.8	6.5
Median score	6.0	6.0	7.0	7.0	6.0	
MADM	1.7	1.3	1.4	1.6	1.6	
Agreement	Low	Moderate	Low	Low	Low	
	No consensus	No consensus	No consensus	No consensus	No consensus	
**Round 2**						

Mean score	6.7	**7.4**	7.0	6.4	5.8	6.7
Median score	6.5	**7.0**	7.0	6.0	6.0	
MADM	1.3	**0.7**	0.7	1.1	1.6	
Agreement	Moderate	**High**	High	Moderate	Low	
	No consensus	**Consensus**	No consensus	No consensus	No consensus	
**Round 3**						
Mean score	6.7	**7.4**	**7.4**	6.4	5.8	6.8
Median score	6.0	**7.0**	**8.0**	6.0	6.0	
MADM	1.0	**0.7**	**0.6**	1.1	1.4	
Agreement	High	**High**	**High**	Moderate	Moderate	
	No consensus	**Consensus**	**Consensus**	No consensus	No consensus	
**99- Child activity preference or perception**						
**Round 1**						
Mean score	6.9	6.8	6.7	6.1	5.9	6.5
Median score	7.0	6.0	7.0	6.0	6.0	
MADM	1.0	0.9	1.1	1.5	1.2	
Agreement	High	High	Moderate	Low	Moderate	
	No consensus	No consensus	No consensus	No consensus	No consensus	
**Round 2**						
Mean score	6.8	7.2	6.5	6.2	6.0	6.5
Median score	7.0	7.0	6.0	6.0	6.0	
MADM	0.9	1.4	0.8	1.1	1.1	
Agreement	High	Moderate	High	High	Moderate	
	No consensus	No consensus	No consensus	No consensus	No consensus	
**Round 3**						
Mean score	7.0	7.9	7.3	6.6	6.0	7.0
Median score	7.0	8.0	8.0	7.0	6.0	
MADM	0.9	1.0	1.0	0.9	1.2	
Agreement	High	High	High	High	Moderate	

	No consensus	No consensus	No consensus	No consensus	No consensus	
**100- Child food preference**						
**Round 1**						
Mean score	7.1	6.7	6.8	6.2	6.2	6.6
Median score	7.0	6.0	7.0	6.0	6.0	
MADM	1.2	1.1	1.0	1.4	1.2	
Agreement	Moderate	High	High	Moderate	Moderate	
	No consensus	No consensus	No consensus	No consensus	No consensus	
**Round 2**						
Mean score	6.9	7.0	6.8	6.1	6.2	6.6
Median score	7.0	6.0	7.0	6.0	6.0	
MADM	1.3	1.0	0.9	1.1	1.1	
Agreement	Moderate	High	High	High	Moderate	
	No consensus	No consensus	No consensus	No consensus	No consensus	
**Round 3**						
Mean score	7.5	7.4	6.2	6.4	6.2	6.7
Median score	7.0	8.0	6.0	6.0	6.0	
MADM	1.3	1.1	0.8	1.3	1.1	
Agreement	Moderate	Moderate	High	Moderate	Moderate	
	No consensus	No consensus	No consensus	No consensus	No consensus	
**101- Child blood pressure**						
**Round 1**						
Mean score	5.4	6.2	6.2	5.0	5.7	5.7
Median score	6.0	7.0	6.0	5.0	6.0	
MADM	1.4	1.1	1.6	1.4	1.5	
Agreement	Moderate	High	Low	Moderate	Low	
	No consensus	No consensus	No consensus	No consensus	No consensus	
**Round 2**						
Mean score	5.6	7.1	6.1	5.0	5.4	5.9

Median score	6.0	7.0	6.0	4.5	5.5	
MADM	1.0	1.0	1.7	1.3	1.5	
Agreement	High	High	Low	Moderate	Low	
	No consensus	No consensus	No consensus	No consensus	No consensus	
**Round 3**						
Mean score	5.8	8.3	6.2	5.0	5.2	6.1
Median score	6.0	8.5	6.0	4.5	5.0	
MADM	0.9	1.3	2.2	1.3	1.4	
Agreement	High	Moderate	Low	Moderate	Low	
	No consensus	No consensus	No consensus	No consensus	No consensus	
**102- Child cholesterol**						
**Round 1**						
Mean score	5.6	6.4	6.0	4.7	5.8	5.7
Median score	6.0	7.0	6.0	5.0	6.0	
MADM	1.6	0.9	1.8	1.4	1.6	
Agreement	Low	High	Low	Low	Low	
	No consensus	No consensus	No consensus	No consensus	No consensus	
**Round 2**						
Mean score	5.7	7.4	5.9	4.8	5.2	5.8
Median score	6.0	7.0	6.0	5.0	5.0	
MADM	1.3	0.7	1.9	1.2	1.5	
Agreement	Moderate	High	Low	Moderate	Low	
	No consensus	No consensus	No consensus	No consensus	No consensus	
**Round 3**						
Mean score	6.0	7.2	5.5	4.9	5.1	5.7
Median score	6.0	7.0	5.0	4.5	5.0	
MADM	1.2	1.0	1.5	1.4	1.4	
Agreement	Moderate	High	Low	Moderate	Moderate	
	No consensus	No consensus	No consensus	No consensus	No consensus	

**103- Child glucose**						
**Round 1**						
Mean score	5.9	6.4	6.8	5.3	5.7	6.0
Median score	6.0	7.0	7.0	5.0	6.0	
MADM	1.6	0.9	1.7	1.4	1.7	
Agreement	Low	High	Low	Moderate	Low	
	No consensus	No consensus	No consensus	No consensus	No consensus	
**Round 2**						
Mean score	6.7	7.0	6.5	5.4	5.5	6.2
Median score	7.0	7.0	7.0	5.0	5.5	
MADM	1.1	0.5	1.7	1.2	1.6	
Agreement	Moderate	High	Low	Moderate	Low	
	No consensus	No consensus	No consensus	No consensus	No consensus	
**Round 3**						
Mean score	6.5	6.8	6.4	5.1	5.4	6.1
Median score	6.0	7.0	7.0	5.0	5.0	
MADM	1.2	0.2	2.2	1.1	1.4	
Agreement	Moderate	High	Low	Moderate	Moderate	
	No consensus	No consensus	No consensus	No consensus	No consensus	
**104- Child hemoglobin**						
**Round 1**						
Mean score	5.5	5.9	5.6	4.8	5.4	5.4
Median score	6.0	7.0	6.0	5.0	5.0	
MADM	1.7	1.3	1.8	1.4	1.7	
Agreement	Low	Moderate	Low	Moderate	Low	
	No consensus	No consensus	No consensus	No consensus	No consensus	
**Round 2**						
Mean score	5.5	7.0	5.4	4.8	5.1	5.6
Median score	6.0	7.0	6.0	5.0	5.0	

MADM	1.5	0.4	1.9	1.3	1.5	
Agreement	Low	High	Low	Moderate	Low	
	No consensus	No consensus	No consensus	No consensus	No consensus	
**Round 3**						
Mean score	5.5	7.0	5.8	5.4	4.7	5.7
Median score	5.0	7.0	5.5	5.0	4.5	
MADM	1.7	0.5	1.8	1.9	1.5	
Agreement	Low	High	Low	Low	Low	
	No consensus	No consensus	No consensus	No consensus	No consensus	
**105- Child lipids**						
**Round 1**						
Mean score	5.4	5.9	5.9	5.1	5.8	5.6
Median score	6.0	7.0	6.0	5.0	6.0	
MADM	1.7	1.3	1.9	1.4	1.0	
Agreement	Low	Moderate	Low	Moderate	High	
	No consensus	No consensus	No consensus	No consensus	No consensus	
**Round 2**						
Mean score	5.7	6.4	5.4	5.2	5.4	5.6
Median score	6.0	7.0	6.0	5.0	5.0	
MADM	1.0	0.6	1.4	1.2	1.6	
Agreement	High	High	Low	Moderate	Low	
	No consensus	No consensus	No consensus	No consensus	No consensus	
**Round 3**						
Mean score	5.8	6.3	5.3	4.8	5.2	5.4
Median score	6.0	6.5	5.5	4.5	5.0	
MADM	0.9	0.8	1.3	1.3	1.5	
Agreement	High	High	Moderate	Moderate	Low	
	No consensus	No consensus	No consensus	No consensus	No consensus	
**106- Child metabolic and inflammatory parameters**						

**Round 1**						
Mean score	5.8	6.3	6.3	4.9	6.3	5.9
Median score	6.0	7.0	6.0	5.0	6.0	
MADM	1.5	1.2	1.7	1.6	1.6	
Agreement	Low	Moderate	Low	Low	Low	
	No consensus	No consensus	No consensus	No consensus	No consensus	
**Round 2**						
Mean score	6.1	6.8	5.6	5.2	5.5	5.9
Median score	6.0	7.0	6.0	5.0	6.0	
MADM	0.9	0.6	1.4	1.2	1.4	
Agreement	High	High	Moderate	Moderate	Low	
	No consensus	No consensus	No consensus	No consensus	No consensus	
**Round 3**						
Mean score	6.2	6.3	5.8	5.1	5.3	5.7
Median score	6.0	7.0	6.5	5.0	5.5	
MADM	0.6	0.7	1.3	1.1	1.5	
Agreement	High	High	Moderate	Moderate	Low	
	No consensus	No consensus	No consensus	No consensus	No consensus	
**107- Child health-related quality of life**						
**Round 1**						
Mean score	7.5	**7.7**	7.5	6.8	6.8	7.2
Median score	8.0	**8.0**	7.5	7.0	7.0	
MADM	1.3	**0.7**	1.2	1.4	1.3	
Agreement	Moderate	**High**	Moderate	Moderate	Moderate	
	No consensus	**Consensus**	No consensus	No consensus	No consensus	
**Round 2**						
Mean score	**7.6**	**7.8**	7.2	7.0	6.9	7.3
Median score	**8.0**	**8.0**	7.0	7.0	7.0	
MADM	**0.9**	**0.8**	0.9	1.3	1.1	

Agreement	**High**	**High**	High	Moderate	Moderate	
	**Consensus**	**Consensus**	No consensus	No consensus	No consensus	
**Round 3**						
Mean score	**7.7**	**8.1**	**7.7**	7.3	7.0	7.6
Median score	**7.0**	**8.0**	**7.5**	7.5	7.0	
MADM	**0.8**	**0.4**	**1.0**	1.3	1.0	
Agreement	**High**	**High**	**High**	Moderate	High	
	**Consensus**	**Consensus**	**Consensus**	No consensus	No consensus	
**108- Economic evaluation**						
**Round 1**						
Mean score	6.8	6.1	6.3	6.9	6.9	6.6
Median score	7.0	6.0	7.0	7.5	7.0	
MADM	1.5	1.2	1.4	1.6	1.2	
Agreement	Low	Moderate	Moderate	Low	Moderate	
	No consensus	No consensus	No consensus	No consensus	No consensus	
**Round 2**						
Mean score	6.9	6.9	6.2	7.1	7.0	6.8
Median score	7.0	7.0	6.0	7.0	7.0	
MADM	1.1	0.6	0.9	1.1	0.9	
Agreement	Moderate	High	High	High	High	
	No consensus	No consensus	No consensus	No consensus	No consensus	
**Round 3**						
Mean score	7.3	7.1	6.2	6.9	7.1	6.9
Median score	8.0	7.0	6.0	7.0	7.0	
MADM	0.9	0.7	0.6	0.6	0.9	
Agreement	High	High	High	High	High	
	No consensus	No consensus	No consensus	No consensus	No consensus	
**109- Child healthcare utilisation**						
**Round 1**						

Mean score	6.9	6.3	6.4	6.2	6.1	6.4
Median score	7.0	7.0	7.0	6.0	6.0	
MADM	1.4	1.2	1.5	1.4	1.4	
Agreement	Moderate	Moderate	Low	Moderate	Low	
	No consensus	No consensus	No consensus	No consensus	No consensus	
**Round 2**						
Mean score	6.8	6.4	5.8	5.9	6.2	6.2
Median score	7.0	6.0	6.0	6.0	6.0	
MADM	1.1	0.9	0.8	0.9	1.2	
Agreement	Moderate	High	High	High	Moderate	
	No consensus	No consensus	No consensus	No consensus	No consensus	
**Round 3**						
Mean score	7.0	6.5	6.0	5.9	6.2	6.3
Median score	7.0	6.5	6.0	6.0	6.0	
MADM	1.3	0.8	0.7	0.8	1.2	
Agreement	Moderate	High	High	High	Moderate	
	No consensus	No consensus	No consensus	No consensus	No consensus	
**110- Intervention cost**						
**Round 1**						
Mean score	6.8	6.6	6.3	**7.7**	6.9	6.8
Median score	7.0	7.0	6.0	**8.0**	7.0	
MADM	1.6	1.2	1.5	**1.2**	1.1	
Agreement	Low	Moderate	Low	**Moderate**	Moderate	
	No consensus	No consensus	No consensus	**Consensus**	No consensus	
**Round 2**						
Mean score	7.1	**7.4**	6.3	**8.0**	7.0	7.2
Median score	7.0	**7.0**	6.0	**9.0**	7.0	
MADM	1.2	**0.9**	1.1	**1.0**	0.9	
Agreement	Moderate	**High**	Moderate	**High**	High	

	No consensus	**Consensus**	No consensus	**Consensus**	No consensus	
**Round 3**						
Mean score	**7.6**	7.1	6.7	**7.8**	**7.2**	7.3
Median score	**8.0**	7.0	7.0	**8.0**	**7.0**	
MADM	**0.9**	0.7	1.0	**1.2**	**0.8**	
Agreement	**High**	High	High	**Moderate**	**High**	
	**Consensus**	No consensus	No consensus	**Consensus**	**Consensus**	
**111- Child oral hygiene**						
**Round 1**						
Mean score	6.4	6.7	5.9	6.1	5.5	6.1
Median score	6.0	7.0	6.0	6.0	6.0	
MADM	1.7	1.4	1.4	1.6	1.6	
Agreement	Low	Moderate	Moderate	Low	Low	
	No consensus	No consensus	No consensus	No consensus	No consensus	
**Round 2**						
Mean score	6.8	6.9	6.3	5.5	5.5	6.2
Median score	7.0	7.0	6.5	6.0	6.0	
MADM	1.4	1.0	1.5	1.4	1.6	
Agreement	Moderate	High	Low	Moderate	Low	
	No consensus	No consensus	No consensus	No consensus	No consensus	
**Round 3**						
Mean score	7.3	**7.5**	**7.0**	5.8	5.5	6.6
Median score	8.0	**7.0**	**7.0**	6.0	5.0	
MADM	1.4	**0.5**	**0.8**	1.6	1.5	
Agreement	Moderate	**High**	**High**	Low	Low	
	No consensus	**Consensus**	**Consensus**	No consensus	No consensus	
**112- Child dental caries**						
**Round 1**						
Mean score	6.9	6.8	6.0	6.2	5.7	6.3

Median score	7.0	7.0	6.0	6.0	6.0	
MADM	1.6	1.6	1.4	1.7	1.7	
Agreement	Low	Low	Moderate	Low	Low	
	No consensus	No consensus	No consensus	No consensus	No consensus	
**Round 2**						
Mean score	7.1	6.9	6.7	5.8	5.6	6.4
Median score	7.0	6.5	7.0	6.0	6.0	
MADM	1.3	1.3	1.2	1.3	1.5	
Agreement	Moderate	Moderate	Moderate	Moderate	Low	
	No consensus	No consensus	No consensus	No consensus	No consensus	
**Round 3**						
Mean score	**7.5**	**7.8**	**7.2**	5.8	5.7	6.8
Median score	**8.0**	**7.0**	**7.0**	6.0	6.0	
MADM	**1.3**	**0.8**	**1.0**	1.8	1.4	
Agreement	**Moderate**	**High**	**High**	Low	Low	
	**Consensus**	**Consensus**	**Consensus**	No consensus	No consensus	
**113- Birth weight adjusted for gestational age**						
**Round 1**						
Mean score	N/A	N/A	N/A	N/A	N/A	N/A
Median score	N/A	N/A	N/A	N/A	N/A	
MADM	N/A	N/A	N/A	N/A	N/A	
Agreement	N/A	N/A	N/A	N/A	N/A	
	N/A	N/A	N/A	N/A	N/A	
**Round 2**						
Mean score	6.4	5.3	5.9	6.4	6.1	6.0
Median score	6.0	6.0	6.0	7.0	6.0	
MADM	1.2	1.0	0.8	1.2	1.3	
Agreement	Moderate	High	High	Moderate	Moderate	
	No consensus	No consensus	No consensus	No consensus	No consensus	

**Round 3**						
Mean score	6.5	5.6	**6.8**	6.4	5.9	6.2
Median score	7.0	6.0	**7.0**	7.0	6.0	
MADM	1.1	0.8	**0.2**	1.0	1.3	
Agreement	Moderate	High	**High**	High	Moderate	
	No consensus	No consensus	**Consensus**	No consensus	No consensus	
**114- Household Chaos**						
**Round 1**						
Mean score	N/A	N/A	N/A	N/A	N/A	N/A
Median score	N/A	N/A	N/A	N/A	N/A	
MADM	N/A	N/A	N/A	N/A	N/A	
Agreement	N/A	N/A	N/A	N/A	N/A	
	N/A	N/A	N/A	N/A	N/A	
**Round 2**						
Mean score	**7.2**	**7.0**	6.1	6.4	6.4	6.6
Median score	**7.0**	**7.0**	6.0	6.5	6.5	
MADM	**0.9**	**0.4**	1.3	1.8	1.4	
Agreement	**High**	**High**	Moderate	Low	Moderate	
	**Consensus**	**Consensus**	No consensus	No consensus	No consensus	
**Round 3**						
Mean score	**7.3**	**7.2**	6.6	6.4	6.4	6.8
Median score	**8.0**	**7.0**	6.0	6.5	6.0	
MADM	**0.9**	**0.2**	1.0	1.6	1.3	
Agreement	**High**	**High**	High	Low	Moderate	
	**Consensus**	**Consensus**	No consensus	No consensus	No consensus	
**115- Parent child child mutually responsive orientation**						
**Round 1**						
Mean score	N/A	N/A	N/A	N/A	N/A	N/A
Median score	N/A	N/A	N/A	N/A	N/A	

MADM	N/A	N/A	N/A	N/A	N/A	
Agreement	N/A	N/A	N/A	N/A	N/A	
	N/A	N/A	N/A	N/A	N/A	
**Round 2**						
Mean score	7.2	7.1	5.8	5.5	5.5	6.2
Median score	7.0	7.0	5.5	6.0	6.0	
MADM	1.0	0.5	1.2	1.2	1.3	
Agreement	High	High	Moderate	Moderate	Moderate	
	No consensus	No consensus	No consensus	No consensus	No consensus	
**Round 3**						
Mean score	7.4	**7.2**	**6.8**	6.1	5.5	6.6
Median score	8.0	**7.0**	**7.0**	6.0	5.0	
MADM	0.9	**0.2**	**0.5**	1.0	1.3	
Agreement	High	**High**	**High**	High	Moderate	
	No consensus	**Consensus**	**Consensus**	No consensus	No consensus	
**116- Parent/caregiver body image and satisfaction**						
**Round 1**						
Mean score	N/A	N/A	N/A	N/A	N/A	N/A
Median score	N/A	N/A	N/A	N/A	N/A	
MADM	N/A	N/A	N/A	N/A	N/A	
Agreement	N/A	N/A	N/A	N/A	N/A	
	N/A	N/A	N/A	N/A	N/A	
**Round 2**						
Mean score	6.7	**7.1**	6.4	5.5	5.8	6.3
Median score	7.0	**7.0**	7.0	6.0	6.0	
MADM	0.7	**0.6**	1.0	1.0	1.1	
Agreement	High	**High**	High	High	Moderate	
	No consensus	**Consensus**	No consensus	No consensus	No consensus	
**Round 3**						

Mean score	6.7	**7.6**	6.8	5.6	5.8	6.5
Median score	7.0	**8.0**	7.0	6.0	6.0	
MADM	0.9	**0.4**	0.6	0.9	1.2	
Agreement	High	**High**	High	High	Moderate	
	No consensus	**Consensus**	No consensus	No consensus	No consensus	
**117- Child self esteem**						
**Round 1**						
Mean score	N/A	N/A	N/A	N/A	N/A	N/A
Median score	N/A	N/A	N/A	N/A	N/A	
MADM	N/A	N/A	N/A	N/A	N/A	
Agreement	N/A	N/A	N/A	N/A	N/A	
	N/A	N/A	N/A	N/A	N/A	
**Round 2**						
Mean score	7.0	**7.2**	**7.2**	6.2	6.1	6.7
Median score	7.0	**7.0**	**8.0**	6.0	6.0	
MADM	1.0	**0.7**	**1.2**	1.5	1.3	
Agreement	High	**High**	**Moderate**	Low	Moderate	
	No consensus	**Consensus**	**Consensus**	No consensus	No consensus	
**Round 3**						
Mean score	6.5	**7.5**	**8.0**	6.6	5.9	6.9
Median score	7.0	**7.5**	**8.0**	7.0	6.0	
MADM	1.3	**0.8**	**0.3**	1.6	1.1	
Agreement	Moderate	**High**	**High**	Low	Moderate	
	No consensus	**Consensus**	**Consensus**	No consensus	No consensus	
**119- Child fruit intake**						
**Round 1**						
Mean score	N/A	N/A	N/A	N/A	N/A	N/A
Median score	N/A	N/A	N/A	N/A	N/A	
MADM	N/A	N/A	N/A	N/A	N/A	

Agreement	N/A	N/A	N/A	N/A	N/A	
	N/A	N/A	N/A	N/A	N/A	
**Round 2**						
Mean score	6.6	**7.4**	**7.2**	7.4	6.7	7.1
Median score	6.5	**7.0**	**7.0**	7.0	7.0	
MADM	1.2	**0.4**	**0.8**	0.9	1.1	
Agreement	Moderate	**High**	**High**	High	Moderate	
	No consensus	**Consensus**	**Consensus**	No consensus	No consensus	
**Round 3**						
Mean score	6.8	**7.5**	**8.0**	7.3	6.6	7.2
Median score	7.0	**7.0**	**8.0**	7.0	7.0	
MADM	0.7	**0.5**	**0.7**	1.0	1.1	
Agreement	High	**High**	**High**	High	Moderate	
	No consensus	**Consensus**	**Consensus**	No consensus	No consensus	
**120- Child vegetable intake**						
**Round 1**						
Mean score	N/A	N/A	N/A	N/A	N/A	N/A
Median score	N/A	N/A	N/A	N/A	N/A	
MADM	N/A	N/A	N/A	N/A	N/A	
Agreement	N/A	N/A	N/A	N/A	N/A	
	N/A	N/A	N/A	N/A	N/A	
**Round 2**						
Mean score	**7.72**	**8.00**	**8.00**	7.77	7.14	7.7
Median score	**8.00**	**8.00**	**8.00**	9.00	7.00	
MADM	**1.10**	**0.80**	**0.80**	1.23	0.98	
Agreement	**Moderate**	**High**	**High**	Moderate	High	
	**Consensus**	**Consensus**	**Consensus**	No consensus	No consensus	
**Round 3**						
Mean score	**7.9**	**8.3**	**8.2**	**7.9**	7.1	7.9

Median score	**8.0**	**8.5**	**8.0**	**9.0**	7.0	
MADM	**0.8**	**0.7**	**0.5**	**1.1**	1.1	
Agreement	**High**	**High**	**High**	**Moderate**	Moderate	
	**Consensus**	**Consensus**	**Consensus**	**Consensus**	No consensus	

Supplementary Table S5 - Percentage of stakeholders who scored the outcome as critical (score of 7–9) for inclusion, Round 3 of the eDelphi survey

Outcome	Health professionals (%)	Community or organisational (%)	Parents/caregivers (%)	Policy-makers/funders (%)	Researchers (%)
Child weight based anthropometry	93	86	67	90	90
Child body composition	60	57	50	60	73
Child circumference	53	29	50	50	61
Child screen time	80	100	83	90	82
Sedentary behaviour or physical activity home environment	76	100	100	80	78
Child time spent sedentary	94	100	100	100	86
Child active transport	67	83	67	60	59
Child physical fitness	67	83	83	70	51
Child active indoor play	47	83	83	60	55
Child active outdoor play	73	100	100	60	80
Child physical activity	87	100	100	90	96
Infant tummy time	60	67	83	50	49
Infant floor based play time	67	83	100	40	47
Child enjoyment of physical activity and playing	80	100	83	60	45
Child sleep duration	63	67	100	60	69
Child sleep disturbance or problems	69	67	50	40	43
Child sleep efficiency	44	50	83	40	31
Child sleep latency	31	50	50	20	18
Child sleep patterns or timing	69	33	50	40	53
Child sleep quality	81	83	67	60	61
Child carbohydrate intake or glycemic load	53	67	33	30	18
Child dairy intake	60	67	33	40	16
Child diet quality	100	100	100	100	92

Child dietary intake	100	100	100	100	88
Child fat intake	40	83	50	60	31
Child fibre intake	67	83	50	30	33
Child fruit and vegetable intake	93	100	100	70	86
Child grains; breads; cereals intake	47	83	50	50	29
Child meat; poultry; fish intake	33	67	50	60	18
Child non-core food intake	87	67	83	80	69
Child non-core beverages intake	93	83	83	90	84
Child out of home foods intake	80	83	67	90	55
Child protein intake	53	67	67	30	22
Child sodium intake	53	83	33	60	24
Child total and added sugars intake	100	67	100	80	69
Child desire for drinks	60	83	50	30	31
Child eating in response to emotion	80	83	67	70	41
Child enjoyment of food	47	100	67	70	31
Child meal patterns	80	83	83	80	65
Child food neophobia	67	83	50	30	27
Child satiety responsiveness	80	83	83	70	47
Child slowness in eating	20	67	17	30	14
Parent/caregiver weight based anthropometry	69	83	60	50	68
Parent/caregiver screen time parenting practice	69	86	100	60	68
Parent/caregiver role modelling of sedentary behaviours	88	100	100	50	64
Parent/caregiver role modelling of physical activity	94	100	100	70	82
Parent/caregiver role modelling of healthy eating	100	100	100	80	86
Parent/caregiver encouragement of physical activity	94	100	80	70	76
Parent/caregiver co-participation in physical activity	87	100	80	70	66

Parent/caregiver physical activity parenting practice	75	100	100	70	56
Parent/caregiver sleep parenting practice	60	71	60	40	42
Parent/caregiver daytime dysfunction or fatigue	60	71	60	20	18
Parent/caregiver nutrition parenting practices	93	86	80	60	68
Parent/caregiver's dietary intentions	67	71	60	60	32
Parent/caregiver food variety	73	100	60	60	56
Parent/caregiver anxiety symptoms	67	71	60	50	36
Parent/caregiver concerns about child's weight	53	86	40	60	58
Parent/caregiver depression symptoms	67	71	40	30	53
Parent/caregiver self-efficacy	67	86	60	50	61
Parent/caregiver social support	53	71	80	70	57
Parent/caregiver stress	67	50	60	50	55
Parent/caregiver attitudes	87	100	60	60	47
Parent/caregiver awareness of behaviours	87	100	60	50	45
Parent/caregiver beliefs	87	83	80	50	39
Parent/caregiver health literacy	87	100	80	70	65
Parent/caregiver knowledge	87	100	60	60	31
Parent/caregiver readiness to change	80	100	60	60	49
Parenting style	87	100	40	60	41
Parent/caregiver responsiveness	87	83	60	50	47
Family functioning	73	83	60	70	47
Parent/caregiver perception of child weight	73	83	60	70	61
Perception of parent/caregiver influence on child's health behaviours	87	100	60	60	53
Parent/caregiver perceptions of adequacy of resources and barriers	87	100	60	90	57
Parent/caregiver feeding control	88	86	17	70	59
Parent/caregiver emotional feeding	87	86	17	60	51

Parent/caregiver feeding encouragement	60	86	50	70	55
Parent/caregiver feeding interaction	80	100	50	70	55
Parent/caregiver feeding structure	67	100	33	60	47
Parent/caregiver feeding style	67	100	50	70	53
Parent/caregiver using food as a reward	93	86	50	70	69
Parent/caregiver frequency of bottle use	80	57	17	70	35
Parent/caregiver promoting autonomy	73	86	83	60	59
Parent/caregiver that is responsible for feeding	80	86	50	60	37
Food environment	88	100	100	90	92
Household food security	93	100	100	90	90
Family meal environment	88	100	100	70	88
Early childhood education and care (ECEC) environment	69	100	83	90	69
Healthcare environment	38	83	67	50	27
Child internalising and/or externalising behaviours	47	67	50	10	20
Child emotion regulation	67	67	67	40	31
Child emotional development	53	67	83	30	22
Child school readiness	27	17	50	30	20
Child wellbeing	87	83	100	90	65
Child attention control	53	33	33	20	10
Child cognitive development	47	67	50	30	20
Child executive function	33	67	67	20	24
Child self-regulation	60	83	33	60	51
Child fundamental movement skills	40	86	80	40	33
Child activity preference or perception	60	71	67	60	35
Child food preference	67	57	33	40	35
Child blood pressure	13	50	33	10	18
Child cholesterol	25	67	17	10	20
Child glucose	38	67	50	10	31

Child hemoglobin	40	50	17	30	14
Child lipids	20	33	17	10	27
Child metabolic and inflammatory parameters	33	33	33	10	22
Child health-related quality of life	93	100	83	60	70
Economic evaluation	73	71	33	73	71
Child healthcare utilisation	63	43	33	27	65
Intervention cost	81	71	67	80	81
Child oral hygiene	67	100	80	40	39
Child dental caries	87	83	80	40	56
Birth weight adjusted for gestational age	60	17	80	60	57
Household Chaos	75	100	33	40	62
Parent child child mutually responsive orientation	60	100	83	43	49
Parent/caregiver body image and satisfaction	73	83	60	20	52
Child self-esteem	60	83	100	50	62
Child fruit intake	60	100	83	70	69
Child vegetable intake	93	100	100	80	71

Supplementary Figure S1 – Example of graphical feedback given to participants between rounds

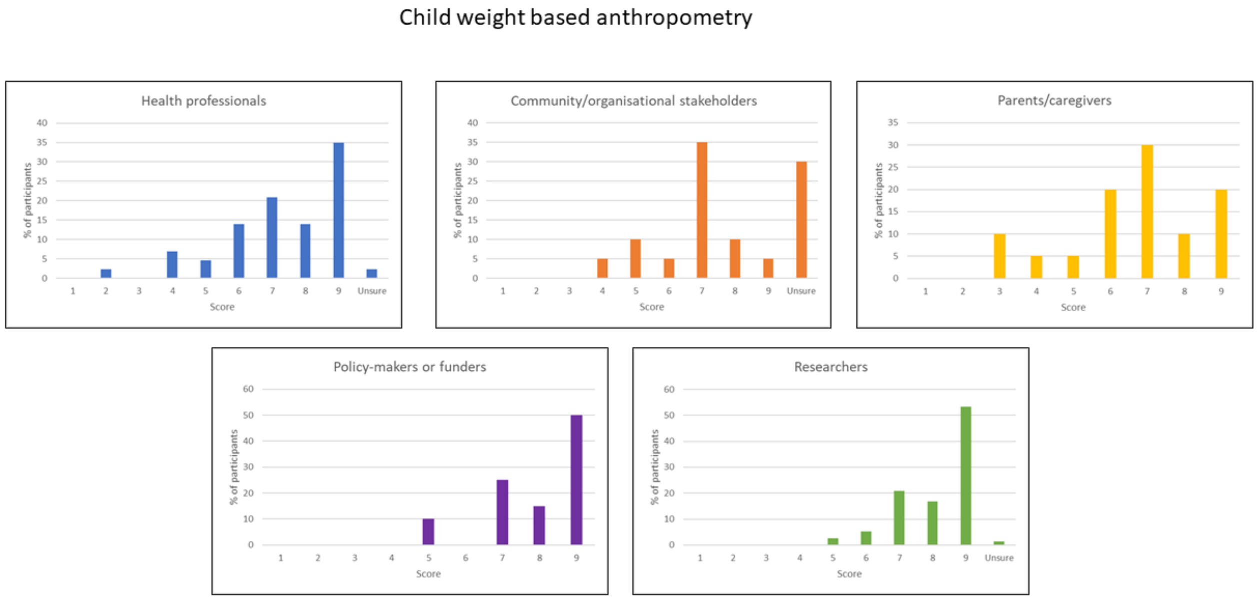

Supplementary Figure S2 - Distribution of scores across all outcomes

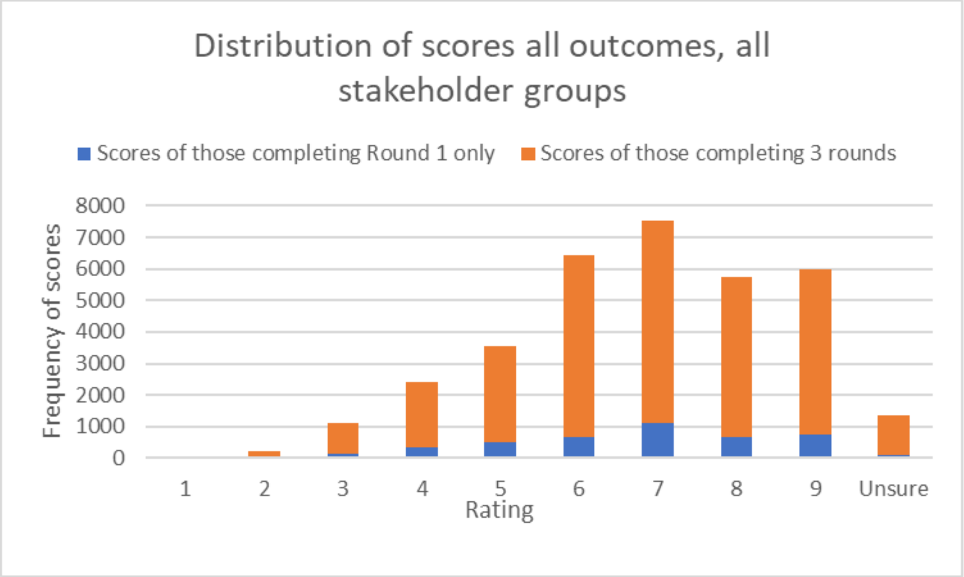

Figure notes: Blue bars represent those participants who scored only in Round 1, orange bars represent those participants who scored in all rounds

REFERENCES

1. Kirkham JJ, Gorst S, Altman DG, Blazeby JM, Clarke M, Devane D, et al. Core Outcome Set–STAndards for Reporting: The COS-STAR Statement. PLoS Med. 2016;13(10):e1002148.
2. Jünger S, Payne SA, Brine J, Radbruch L, Brearley SG. Guidance on Conducting and REporting DElphi Studies (CREDES) in palliative care: Recommendations based on a methodological systematic review. Palliat Med. 2017;31(8):684-706.
